# DUSP22 inhibits lung tumorigenesis by suppression of EGFR/c-Met signaling

**DOI:** 10.1038/s41420-024-02038-8

**Published:** 2024-06-14

**Authors:** Hsiao-Han Lin, Cheng-Wei Chang, Yu-Ting Liao, Shauh-Der Yeh, Hsiu-Ping Lin, Hui-Min Ho, Chantal Hoi-Yin Cheung, Hsueh-Fen Juan, Yi-Rong Chen, Yu-Wen Su, Li-Mei Chen, Tse-Hua Tan, Wen-Jye Lin

**Affiliations:** 1 Immunology Research Center, National Health Research Institutes, Miaoli County, 35053 Taiwan; 2 Department of Urology, Graduate Institute of Clinical Medicine, Taipei Medical University, Taipei, 110301 Taiwan; 3 Department of Life Science, National Taiwan University, Taipei, 10617 Taiwan; 4 Institute of Molecular and Genomic Medicine, National Health Research Institutes, Miaoli County, 35053 Taiwan

**Keywords:** Non-small-cell lung cancer, Cell migration, Kinases, Cell growth

## Abstract

DUSP22, an atypical dual-specificity phosphatase enzyme, plays a significant role in regulating multiple kinase signaling pathways by dephosphorylation. Our study demonstrated that decreased DUSP22 expression is associated with shorter disease-free survival, advanced TNM (tumor, lymph nodes, and metastasis), cancer stage, and higher tumor grade in lung adenocarcinoma (LUAD) patients. Exogenous DUSP22 expression reduces the colony-forming capacity of lung cancer cells and inhibits xenograft tumor growth primarily by targeting EGFR and suppressing its activity through dephosphorylation. Knockdown of DUSP22 using shRNA enhances EGFR dependency in HCC827 lung cancer cells and increases sensitivity to gefitinib, an EGFR inhibitor. Consistently, genetic deletion of DUSP22 enhances EGFRdel (exon 19 deletion)-driven lung tumorigenesis and elevates EGFR activity. Pharmacological inhibition of DUSP22 activates EGFR, ERK1/2, and upregulates downstream PD-L1 expression. Additionally, lentiviral deletion of DUSP22 by shRNA enhances lung cancer cell migration through EGFR/c-Met and PD-L1-dependent pathways. Gefitinib, an EGFR inhibitor, mechanistically suppresses migration induced by DUSP22 deletion and inhibits c-Met activity. Furthermore, cabozantinib, a c-Met inhibitor, reduces migration and attenuates EGFR activation caused by DUSP22 deletion. Collectively, our findings support the hypothesis that loss of DUSP22 function in lung cancer cells confers a survival advantage by augmenting EGFR signaling, leading to increased activation of downstream c-Met, ERK1/2, and PD-L1 axis, ultimately contributing to the progression of advanced lung cancer.

## Introduction

Genetic alterations in the EGFR gene lead to abnormal activation of multiple oncogenic signaling pathways, resulting in tumorigenesis and supporting EGFR’s tumor-promoting function in various cancers [1–4]. Studies have shown that transgenic expression of EGFR mutants in mouse lung tissue induces the development of lung adenocarcinoma, and treatment with EGFR-targeted tyrosine kinase inhibitors (TKIs) leads to significant tumor regression [5], indicating that activating mutations in the EGFR tyrosine kinase domain increase EGFR activity and accelerate lung tumorigenesis. Interestingly. prolonged activation of EGFR enhances the dependence of lung tumor cells on EGFR signaling [6]. Additionally, several members of the EGFR family are often co-expressed with the RTK c-Met in various human cancers, and c-Met supports the proliferation and survival of non-small cell lung cancer (NSCLC) cells [7]. Increased EGFR/c-Met signaling in lung cancer patients is associated with shorter survival, suggesting that the interplay between EGFR and c-Met signaling plays a crucial role in lung tumor progression and development of resistance to TKI treatments [8].

Recently, a group of dual-specificity phosphatases (DUSPs) has emerged as critical regulators of multiple signaling pathways by dephosphorylating and thereby inactivating or activating MAPKs [9]. The majority of research has focused on understanding how aberrant MAPK signaling facilitates the development and progression of cancer [10]. Additionally, malfunctioning phosphatases contribute to tumorigenesis and resistance by allowing uncontrolled kinase activation [11]. For example, loss of phosphatase and tensin homolog (PTEN) expression in lung cancer cells leads to resistance to the TKI erlotinib by activating EGFR and Akt kinases [12]. We postulated that when negative regulators, such as inhibitory phosphatases of EGFR, are lost, it leads to abnormal activation of EGFR for amplifying EGFR-driven oncogenic processes. DUSP22 (also referred to as JKAP or LMW-DSP2) is classified as an atypical member within the DUSP family of phosphatases due to its absence of a MAP kinase-interacting motif [13]. Previous studies have demonstrated that DUSP22 plays a significant role in regulating various signaling proteins, including estrogen receptor α (ERα), MAPKs, STAT3, FAK, and Lck [14–19]. Through dephosphorylation, DUSP22 modulates the activity of these molecules, indicating its ability to govern multiple signaling pathways in different cell types. Abnormal expression of certain DUSPs has been observed in various cancers [20–22]. Notably, studies have revealed down-regulation of DUSP22 expression in breast cancer, anaplastic large cell lymphoma, peripheral T-cell lymphoma, and colorectal cancer [23–26]. Previously, we identified DUSP22 as a negative regulator of the EGFR-androgen receptor (AR) signaling pathway in prostate cancer cells through dephosphorylation [27]. Based on these findings, it is reasonable to explore whether the same regulatory connection between DUSP22 and EGFR plays a significant role in repressing the development and progression of EGFR-driven lung cancer. We hypothesized that DUSP22 is a crucial phosphatase for controlling EGFR signaling and that the loss of DUSP22 function may accelerate the development of EGFR-driven lung tumors.

In this study, we showed that DUSP22 expression was significantly reduced in LUAD tissues compared to non-tumor tissues. Furthermore, we found that DUSP22 expression played a role in suppressing the growth and migration of lung tumor cells by inhibiting EGFR/c-Met signaling and its downstream signaling molecules. Remarkably, our analysis of The Cancer Genome Atlas (TCGA) LUAD datasets using bioinformatics analysis revealed that patients with low DUSP22 expression had poorer disease-free survival. The results support our hypothesis that the phosphatase activity of DUSP22 inhibits EGFR/c-Met signaling and underscore the important role of DUSP22 in suppressing EGFR signaling to prevent lung tumor formation.

## Results

### Low DUSP22 expression in lung cancer tissues of LUAD patients correlates with shorter disease-free survival

**Fig. 1:**
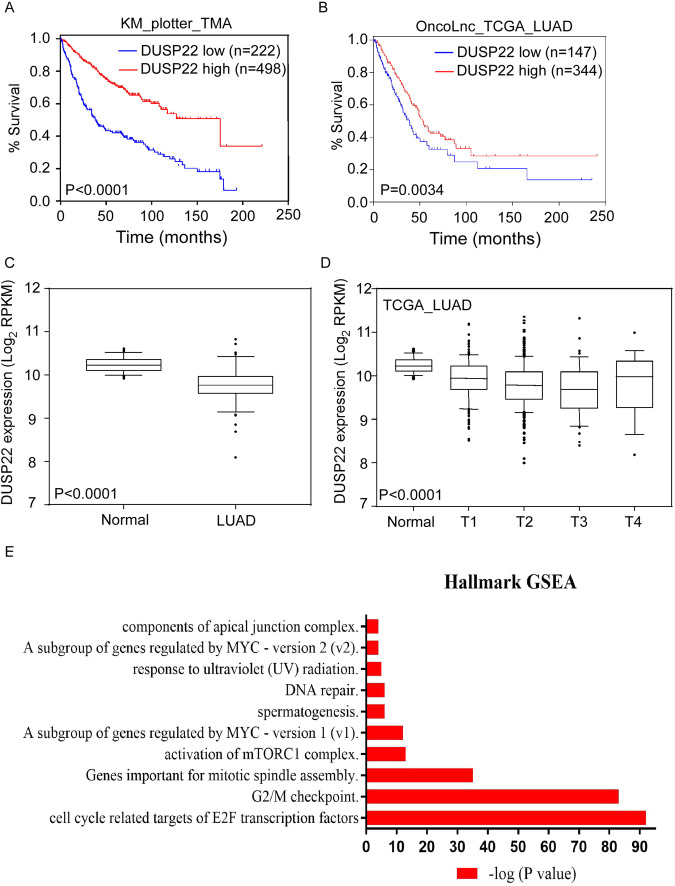
Low DUSP22 expression correlates with poor outcome for LUAD patients. **A** Survival analysis was performed for the meta-analysis of LUAD datasets on KM plotter and **B** for TCGA on OncoLnc according to DUSP22 expression levels. Log rank p values were < 0.0001 and = 0.0034, respectively. **C** DUSP22 expression levels were compared between LUAD and paired adjacent normal tissues in the TCGA LUAD dataset. The box-and-whisker plot shows the 10-90th percentiles with the median represented by the center line. The Wilcoxon rank-sum test was used to determine statistical significance. p < 0.0001. **D** DUSP22 expression levels were compared across normal lung and LUAD at different tumor stages in the TCGA LUAD dataset (one-way ANOVA: p < 0.0001). T represents the size and/or extent of the primary tumor. A higher number following the T indicates a larger tumor or a greater degree of invasion into nearby tissues. **E** Gene Set Enrichment Analysis (GSEA) was performed to determine enriched signaling pathways in LUAD patients with low DUSP22 expression.

We first determined the clinical significance of DUSP22 expression in LUAD patients by analyzing multiple publicly accessible LUAD datasets, including TCGA, using web tools such as the Kaplan-Meier plotter (http://kmplot.com/analysis/) and the OncoLnc web site (http://www.oncolnc.org/) [28–30]. Our data showed that LUAD patients with low DUSP22 expression exhibited notably reduced disease-free survival across multiple LUAD datasets (Fig. 1A, B, and Fig. S1A–C). However, we did not observe a significant correlation between the expression of DUSP22 and the prognosis of lung squamous cell carcinoma (LUSC) patients (Fig. S1D). The expression of DUSP22 exhibited a significant decrease in LUAD tissues when compared to adjacent normal tissues (Fig. 1C). Additionally, low expression of DUSP22 was significantly associated with various clinicopathological parameters, such as tumor stage, TNM cancer stage, and tumor grade (Fig. 1D and Table 1), suggesting that decreased DUSP22 expression could be a key factor in the initiation and advancement of LUAD.

**Table 1:** The correlation between DUSP22 expression and clinicopathological characteristics in LUAD.

Factors	Sample	Univariate analysis^a^			Multivariate analysis^a^		
		HR	95% CI	Wald’s *P* value	HR	95% CI	Wald’s *P* value
Total	344						
DUSP22 expression				**0.021**			**0.041**
High expression	241						
Low expression	103	1.5	1.1–2.1		1.5	1.0–2.1	
Age				0.792			0.147
<65^b^	151						
≥65	193	1.0	0.7–1.5		1.3	0.9–1.9	
Gender				0.528			0.483
Female	174						
Male	170	1.1	0.8–1.6		0.9	0.6–1.2	
pT status							
T1	103						
T2	195	1.8	1.1–2.8	**0.012**	1.6	1.0–2.5	**0.050**
T3	28	4.3	2.3–8.0	**<0.001**	3.6	1.7–7.2	**<0.001**
T4	18	3.5	1.7–7.0	**0.004**	1.6	0.7–3.7	0.223
pN status							
N0	217						
N1	74	2.1	1.4–3.1	**<0.001**	2.3	1.3–4.2	**0.005**
N2 + N3	53	3.2	2.1–4.9	**<0.001**	2.0	0.8–4.7	0.122
pM status							
M0	323						
M1	21	1.9	1.1–3.3	**0.032**			NA^c^
Stage							
I	177						
II	85	1.8	1.2–2.7	**0.008**	0.8	0.4–1.5	0.510
III	61	3.4	2.3–5.2	**<0.001**	1.4	0.5–3.7	0.473
IV	21	2.9	1.6–5.4	**<0.001**	2.0	1.0–4.3	0.066

Univariate and multivariate Cox regression analyses were used for analysis of DUSP22 expression and clinicopathological factors in LUAD patients from TCGA datasets. Bold values indicate statistical significance (p < 0.05).

p: stage given by histopathologic examination of a surgical specimen, *TNM* tumor, node, and metastasis, T1, T2, T3, T4: Refers to the size and/or extent of the main tumor; N0: No cancer in nearby lymph nodes; N1, N2, N3: Refers to the number and location of lymph nodes that contain cancer; M0: Cancer has not spread to other parts of the body. M1: Cancer has spread to other parts of the body. Stage I, II, III, IV: Represents a different level of tumor growth, invasion. *CI* confidence interval, *HR* hazard ratio.

^a^Cox regression model.

^b^The mean age is 65 years for LUAD patients from TCGA. Samples are divided into two groups based on the mean age.

^c^As M1 completely overlapped with stage IV, the P value was not calculated in the Cox regression model.

Next, we conducted gene correlation analysis and Hallmark GSEA with genes that exhibited a negative correlation with DUSP22 expression in the TCGA LUAD patient dataset to identify enriched signaling pathways. The pathways showing the most significant enrichment were marked by increased expression of E2F target genes, progression of the cell cycle, and genes involved in DNA repair (Fig. 1E). We have identified a specific set of cell cycle genes that show a direct correlation with low DUSP22 expression in LUAD patients (Fig. S1E). These cell cycle genes have been linked to unfavorable prognosis outcomes in various types of cancer [31]. Taken together, the results of our bioinformatics analysis indicate that DUSP22 has the potential to serve as a biomarker for predicting disease progression and adverse prognosis in LUAD patients.

### The EGFR signaling pathway is inhibited by DUSP22, leading to the inhibition of lung cancer cell growth

**Fig. 2:**
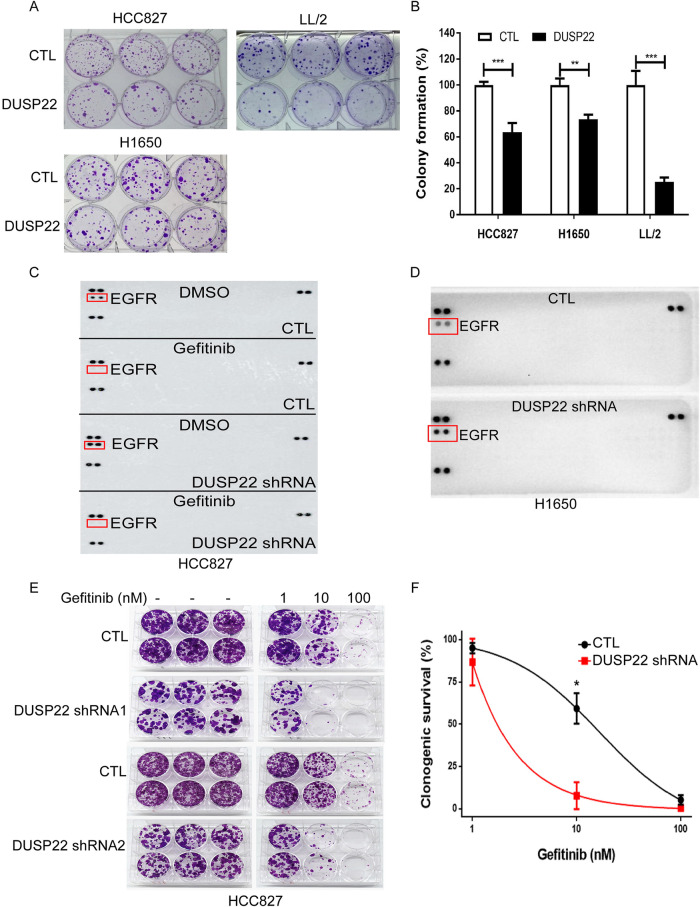
DUSP22 suppresses growth and EGFR activity in several lung cancer cell lines. DUSP22 expression inhibits colony formation in human and murine lung cancer cell lines. **A** Representative images of colony formation in the presence or absence of DUSP22. **B** Statistical results for colony formation replicates (n = 6 per group). **C** A phospho-receptor tyrosine kinase antibody array showed that DUSP22-deleted HCC827 lung cancer cells had increased levels of phosphorylated EGFR, which was reduced with gefitinib treatment. **D** EGFR was the main target kinase with phosphorylation levels that were significantly increased in DUSP22-deleted H1650 lung cancer cells. **E** Representative images of a colony formation assay by crystal violet staining. Cells (HCC827-CTL, HCC827-DUSP22 shRNA1, and HCC827-DUSP22 shRNA2) were plated in 24-well plates in the presence or absence of gefitinib treatment (1, 10, 100 nM) as indicated. HCC827 cells were stained with crystal violets 14 days after treatment. **F** DUSP22 deletion enhanced EGFR addiction in HCC827 cancer cells. DUSP22-deleted HCC827 were highly sensitive to gefitinib at 10 nM. The colony numbers were analyzed and quantified for their survival curve against the EGFR inhibitor, gefitinib at different concentrations as indicated (n = 6 per group). The data are presented as the mean ± SD. Statistical analysis was performed using the ANOVA test. *p < 0.05; **p < 0.01; ***p < 0.001. CTL control.

To investigate how DUSP22 suppresses lung cancer development and progression, we stably introduced wild-type DUSP22 expression into various human and mouse lung cancer cell lines (HCC827, H1650, LL/2, H520, and TC-1) because lung cancer cells typically exhibit low endogenous levels of DUSP22 protein expression (Fig. S2A). Exogenous DUSP22 expression was found to inhibit colony formation in multiple lung cancer cell lines, including HCC827, H1650, and LL/2 (Fig. 2A, B), indicating a suppressive role of DUSP22 in lung tumorigenesis. Considering the dephosphorylation function of DUSP22, we performed a phosphokinase array to identify kinases in both HCC827 (gefitinib-sensitive) and H1650 (gefitinib-resistant) lung cancer cells in the presence or absence of DUSP22 shRNA (± gefitinib for HCC827). EGFR was identified as the primary target of DUSP22 in both HCC827 and H1650 lung cancer cells. (Fig. 2C, D, and Fig. S2B). Importantly, our data revealed a consistent dephosphorylation-based regulation between DUSP22 and EGFR in different lung cancer cell lines harboring wild-type (WT) or mutant EGFR genes (Fig. 2D and Fig. S2C).

To confirm the link between DUSP22-induced growth inhibition and EGFR in lung cancer cells, we assessed the colony-forming capacity of EGFR^low^ H520 and TC-1 cancer cells in the presence or absence of DUSP22 expression (Fig. S2A). Significantly, DUSP22 failed to inhibit colony formation in EGFR^low^ H520 and TC-1 cells (Fig. S2D), suggesting that DUSP22 predominantly acts on EGFR and hinders EGFR signaling to impede lung cancer cell proliferation via dephosphorylation, regardless of their EGFR mutation status. Western blot analysis confirmed that introducing DUSP22 resulted in a significant decrease in pEGFR and pERK1/2 levels in HCC827 and H1650 lung cancer cells (Fig. S2E, F). Notably, DUSP22 expression had a minimal effect on pAkt levels in both cell lines (Fig. S2E, F). These findings provide evidence for the role of DUSP22 in regulating the EGFR/ERK signaling pathway in lung cancer cells harboring constitutively-active EGFR mutations.

Next, we conducted colony formation assays to investigate the impact of DUSP22 deletion on the tumorigenicity of HCC827 lung cancer cells in response to gefitinib. Surprisingly, we observed a notable decrease in the colony forming ability of DUSP22-deleted HCC827 lung cancer cells when treated with gefitinib at a concentration of 10 nM (Fig. 2E, F), suggesting that higher EGFR activity in DUSP22-deleted HCC827 lung cancer cells may make them more vulnerable to EGFR TKI treatment. Unexpectedly, when gefitinib was absent, the deletion of DUSP22 expression had an adverse effect on the colony-forming ability and proliferation of HCC827 cells (Fig. 2E and Fig. S2G), implicating that maintaining functional DUSP22 expression is crucial for regulating the activity of substrate kinases, preventing the occurrence of undesired toxicity caused by the hyperactivation of downstream kinases [32].

### Genetic ablation of DUSP22 in mice results in acceleration of EGFRdel-driven lung tumorigenesis

**Fig. 3:**
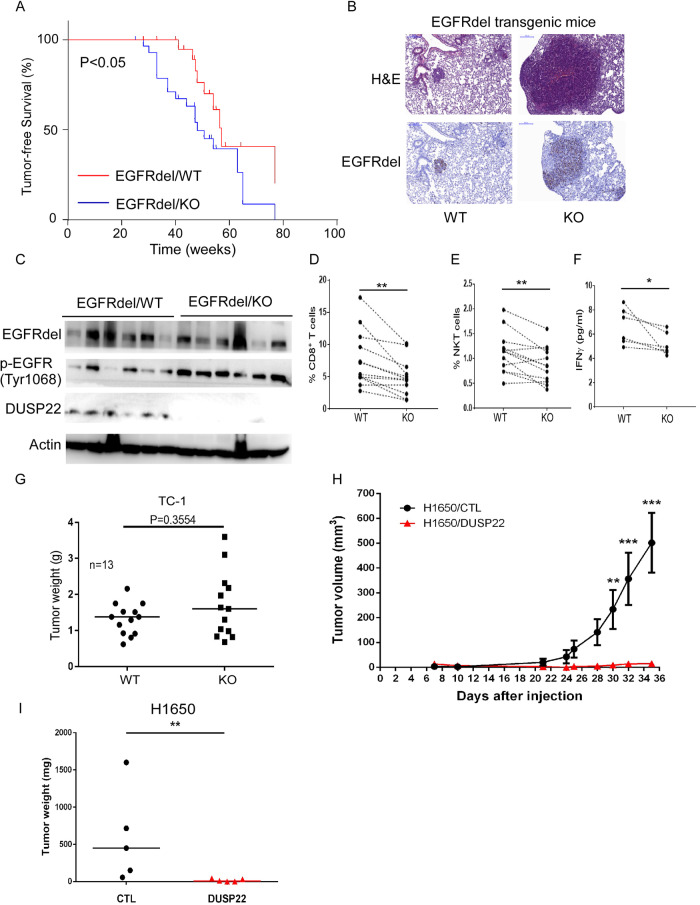
DUSP22 ablation enhances EGFRdel-driven lung tumorigenesis in mice. **A** Tumor-free survival of EGFRdel/DUSP22 WT and EGFRdel/DUSP22 KO mice (n = 26 for EGFRdel/DUSP22 WT and n = 30 for EGFRdel/DUSP22 KO). Statistical analysis was performed using the log-rank test. p value = 0.0354. **B** Representative images of lung tissues from EGFRdel/DUSP22 WT and EGFRdel/DUSP22 KO mice after H&E staining and EGFRdel IHC, respectively. **C** Western blot analysis of mouse lung extracts isolated from EGFRdel/DUSP22 WT and EGFRdel/DUSP22 KO mice. **D**, **E** In mouse EGFRdel transgenic lung tumors, DUSP22 ablation is associated with an immunosuppressive microenvironment. The number of CD8^+^ T cells and NK1.1^+^ NKT cells in EGFRdel/DUSP22 KO lung cancers was reduced. p value = 0.001 and 0.007, respectively; for each group, n = 12. Paired DUSP22 wild-type and knockout littermates were linked by dashed lines. **F** A Bio-Plex cytokine array was used to detect IFNγ in lung extracts isolated from DUSP22 wild-type and knockout littermates. p value = 0.031; n = 6 in each group. Wilcoxon matched-pairs signed rank test was used to analyzed the data of D-F. *p < 0.05; **p < 0.01; ***p < 0.001. **G** TC-1 lung tumor growth in C57BL/6 WT and DUSP22 KO mice. TC-1 cells (5 ×10^5^ cells per mouse) were subcutaneously injected into WT and DUSP22 KO mice (n = 13). After three weeks, tumors were isolated and weighed. Tumor weights are shown, with each dot representing the weight of one tumor from a WT or DUSP22 KO mouse. p = 0.3554. **H**, **I** H1650-derived lung tumor growth, via tumor size/weight measurements, in immunocompromised SCID mice (± DUSP22 expression, n = 5). Statistical comparisons of tumor volume (**H**) were performed using two-way ANOVA and the data are presented as the mean ± SD. The Wilcoxon Rank Sum Test was used for statistical analysis of mouse tumor weights and the data are presented as the median (**G**, **I**). CTL, vector control; *p < 0.05; **p < 0.01; ***p < 0.001.

Our next investigation delved into the role of DUSP22 in EGFRdel-induced lung tumorigenesis in vivo by breeding EGFRdel transgenic mice, where EGFRdel expression is controlled by the lung-specific surfactant protein C (SPC) promoter, with DUSP22 Wild-type (WT) or DUSP22 knockout (KO) mice to generate EGFR-del/DUSP22 WT and EGFR-del/DUSP22 KO mice [19, 33]. During the observation period of 6 to 18 months, we closely monitored the occurrence of lung tumors in mice of both EGFRdel/DUSP22 WT and KO mice. Our findings showed that the absence of DUSP22 led to a significant acceleration in the formation of lung tumors driven by transgenic EGFRdel expression (Fig. 3A, B, and Fig. S3A). We consistently observed increased phosphorylation of EGFR (Tyr1068) in lung tissues of EGFRdel/DUSP22 KO mice compared to those of EGFRdel/DUSP22 WT mice (Fig. 3C). We assessed the effect of DUSP22 deletion on the response of murine lung tumor cells to gefitinib by treating DUSP22 WT and KO mice with gefitinib for 14 days. Western blot analysis of lung tissue lysates showed that gefitinib effectively reduced pEGFR levels in both EGFRdel/DUSP22 KO and EGFRdel/DUSP22 WT mice (Fig. S3B), indicating that DUSP22-deficient lung tumor cells remained sensitive to gefitinib treatment.

Next, we examined the tumor immune microenvironment in EGFRdel/DUSP22 KO mice by comparing lung tumors with EGFRdel/DUSP22 KO and EGFRdel control mice. Our analysis revealed a notable reduction in CD8^+^ T cells, NKT cells, and tumor IFNγ levels in EGFRdel/DUSP22 KO tumors (Figs. 3D, E, F, and S3C). These findings align with a study by Akaby et al., indicating that constitutive EGFR activation in mouse lung tumors correlates with increased immunosuppressive gene signatures [34]. We postulated that the deletion of DUSP22 in the epithelial or stromal compartments of lung tissues in EGFRdel/DUSP22 KO mice may accelerate EGFRdel-driven lung tumorigenesis. To pinpoint the specific compartment responsible for this enhancement, we established a syngeneic lung tumor model using TC-1 cells implanted subcutaneously in WT and DUSP22 KO mice. TC-1 tumor growth in DUSP22-deficient mice showed no significant difference compared to WT mice (Fig. 3G), suggesting that DUSP22 ablation in immune cells or stromal cells does not significantly promote lung tumor growth in vivo. These findings support the idea that tumor-intrinsic DUSP22 function is crucial in suppressing EGFRdel-driven lung tumorigenesis. In a previous study, PTEN deletion in H1650 cancer cells led to continuous Akt activation, providing a growth advantage even under EGFR inhibition [12]. To assess the impact of DUSP22 expression in suppressing the EGFR/ERK signaling pathway on the growth of EGFR-TKI-resistant H1650 cells with sustained Akt activation, we observed a significant inhibitory effect of DUSP22 on H1650 xenograft tumor growth in SCID (severe combined immunodeficiency disease) mice (Fig. 3H, I), suggesting that DUSP22-mediated inhibition of the EGFR pathway at multiple levels could be highly effective against TKI-resistant PTEN-deleted H1650 cells. Collectively, our data support a role for DUSP22 in the suppression of tumor development in lung cancer cells through the dephosphorylation of key target kinases in the EGFR signaling pathway.

### DUSP22 inhibits cancer cell migration through targeting the crosstalk between EGFR and c-Met signaling

**Fig. 4:**
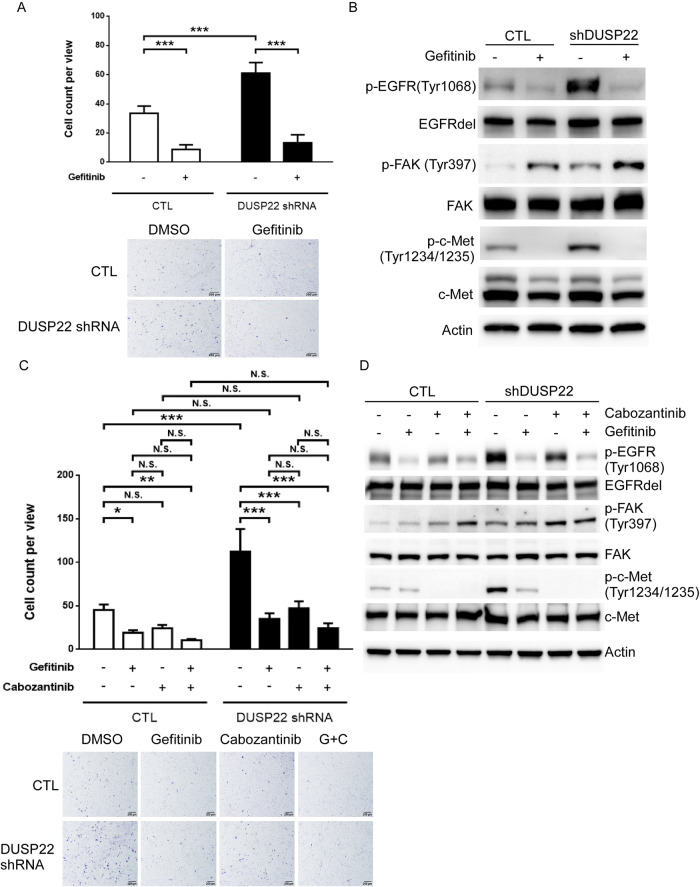
Inhibition of EGFR and c-Met suppresses DUSP22 deletion-induced cell migration in HCC827 cells. **A** CTL and DUSP22-deleted HCC827 cells were treated with vehicle (DMSO) or gefitinib (100 nM) and assessed using the transwell migration assay. Cells that migrated were quantified (upper panel) and photographed (lower panel). Data are representative of three independent experiments (n = 3 per group). **B** Inhibition of EGFR by gefitinib led to downregulation of c-Met activity. HCC827 cells (± DUSP22 shRNA) were treated with DMSO or gefitinib (100 nM). Specific proteins were detected by immunoblotting with the indicated antibodies. **C** Inhibition of EGFR or c-Met function by gefitinib (100 nM) or cabozantinib (5 μM), respectively, repressed cell migration induced by DUSP22 deletion. Migration of cells treated with vehicle, each inhibitor alone, or both inhibitors combined was assessed by transwell migration assay. Cells that migrated were quantified (upper panel) and photographed (lower panel). **D** Phosphorylation levels of EGFR and c-Met were significantly decreased in the presence of gefitinib (100 nM) or cabozantinib (5 μM). CTL and DUSP22-deleted HCC827 cells were treated with DMSO, gefitinib (100 nM), cabozantinib (5 μM), or gefitinib and cabozantinib in combination, then subjected to Western blot analysis by specific antibodies, as indicated. The data are presented as the mean ± SD. Statistical significance was determined by one-way ANOVA. *p < 0.05; **p < 0.01; ***p < 0.001; n.s. not significant.

Prior research has shown that DUSP22 can inhibit the migration of H1299 lung cancer cells with wild-type EGFR by dephosphorylating FAK, a non-receptor tyrosine kinase [18]. We then investigated if deleting DUSP22 would enhance the migration of HCC827 lung cancer cells by boosting EGFR activity. Deletion of DUSP22 using lentiviral shRNA notably increased the migration of HCC827 cells, which was reduced by gefitinib treatment (Fig. 4A). This implies that constitutively-active EGFR mutants can promote lung cancer cell migration, partly through the enhanced EGFR activity resulting from DUSP22 deletion. Additionally, DUSP22 deletion markedly increased p-FAK (Tyr397) levels in HCC827 cells, which were not directly related to cell migration as they persisted at higher levels following gefitinib treatment (Fig. 4B). Importantly, a previous study supports the crucial role of c-Met in cell migration and invasion driven by activated EGFR [8]. We investigated how c-Met affects cell migration in response to DUSP22 deletion using cabozantinib, a c-Met inhibitor [35]. Cabozantinib significantly reduced DUSP22 deletion-induced cell migration (Fig. 4C), highlighting the importance of c-Met in regulating cell migration induced by DUSP22 deletion. Combining gefitinib and cabozantinib did not further suppress DUSP22 deletion-induced cell migration (Fig. 4C), suggesting a potential mutual dependence or redundancy in EGFR and c-Met RTK activation after DUSP22 depletion, jointly controlling cell migration. To explore this further, we showed that gefitinib and cabozantinib individually inhibit EGFR and c-Met activity (Fig. 4D). Gefitinib reduced pc-Met levels, while cabozantinib decreased pEGFR levels in DUSP22-deleted HCC827 cells (Fig. 4D). Cabozantinib effectively inhibited DUSP22 deletion-induced cell migration but increased FAK Tyr397 phosphorylation (Fig. 4D). Cabozantinib did not suppress colony-forming ability in DUSP22-depleted HCC827 cells (Fig. S3D), suggesting a crosstalk between EGFR and c-Met signaling that predominantly facilitates cell migration after DUSP22 deletion in HCC827 cells.

### DUSP22 functions as a negative regulator of PD-L1, a downstream target of EGFR signaling

**Fig. 5:**
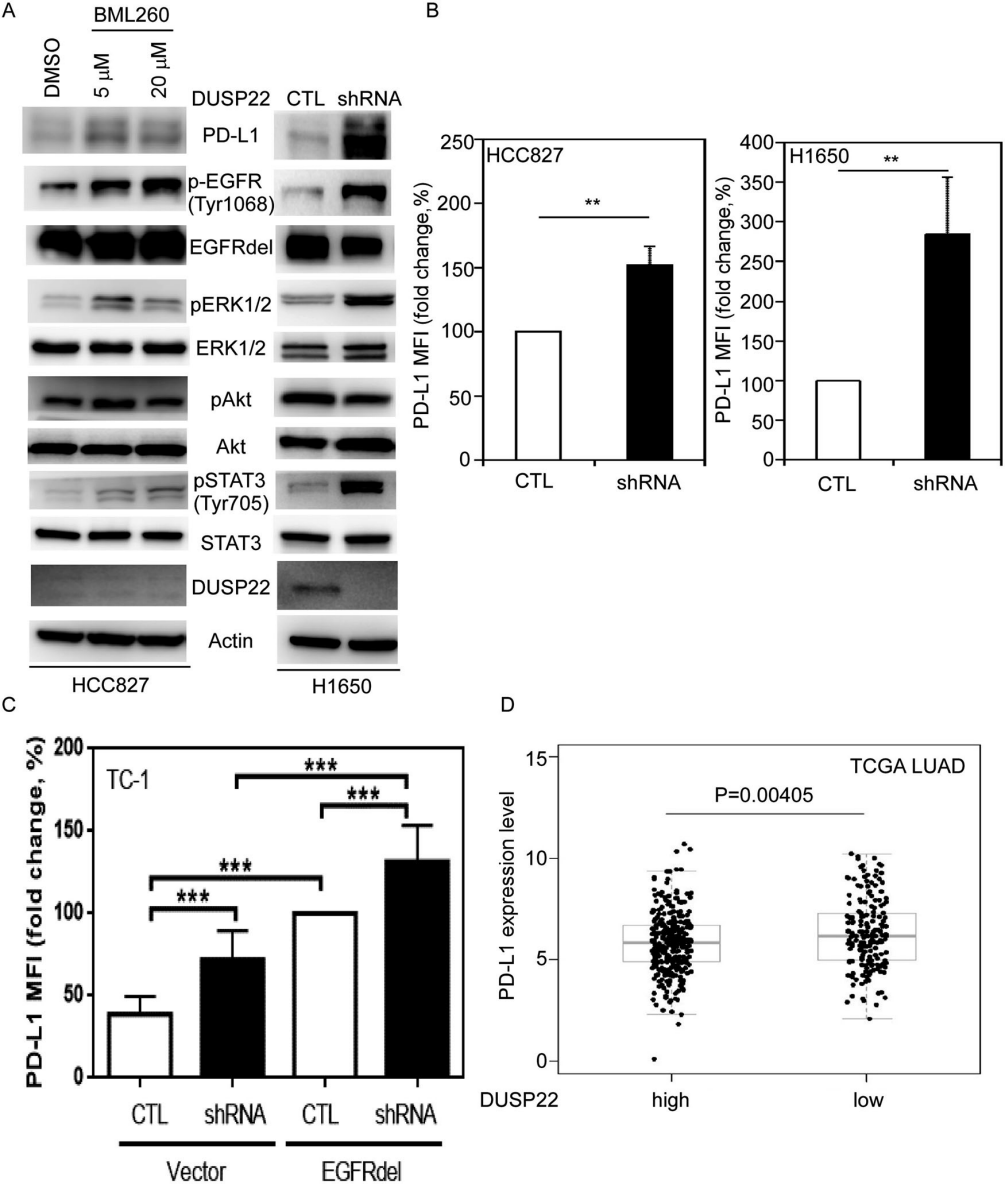
Inhibition of DUSP22 function by a small molecule inhibitor or via specific shRNA increases EGFR signaling and PD-L1 expression. **A** The phosphorylation levels of EGFR, ERK1/2, and STAT3 were markedly increased by BML260 (left panel) or DUSP22 shRNA (right panel) in HCC827 and H1650 cells, respectively. **B** Surface PD-L1 proteins in HCC827 (left panel) and H1650 cells (right panel), in the presence or absence of DUSP22 shRNA, as measured by flow cytometry. Student’s t-test was used for statistical analysis. n = 3 per group. Data are representative of three independent experiments. **C** DUSP22 depletion induced PD-L1 protein upregulation in mouse lung tumor TC-1 cells expressing human EGFRdel mutants and DUSP22-specific shRNA. n = 3 per group. Data are representative of three independent experiments. Statistical significance was determined by one-way ANOVA. **D** Increased PD-L1 expression was associated with LUAD patients with reduced DUSP22 expression in the TCGA LUAD dataset. CTL: vector control. The data are presented as the mean ± SD. Statistical analysis was determined by the Wilcoxon rank-sum test. *p < 0.05; **p < 0.01; ***p < 0.001.

PTEN loss enhances cancer cell immune evasion by upregulating PD-L1 expression [36]. One study demonstrated that mutated EGFR (T790M/L858R)-driven lung tumors can suppress host immunity via PD-1/PD-L1 pathways [34]. The interplay of DUSP22 with EGFR and PD-L1 in lung cancer cells remains unclear. Using a DUSP22 inhibitor, BML260 [37], we examined its impact on EGFR signaling and PD-L1 levels. BML260-mediated inhibition of DUSP22 function increased pEGFR, pERK1/2, pSTAT3, as well as PD-L1 protein expression (Fig. 5A, left panel). Knockdown of DUSP22 by shRNA in H1650 cells similarly elevated levels of these signaling molecules (Fig. 5A, right panel), suggesting a link between DUSP22, EGFR signaling, and PD-L1 expression. In addition, DUSP22 deletion induces PD-L1 upregulation in lung cancer cells, as confirmed by FACS analysis (Fig. 5B).

Upregulated PD-L1 expression was observed in EGFR^low^ TC-1 cell expressing human EGFRdel (Fig. 5C). Knockdown of DUSP22 increased PD-L1 expression in TC-1 cells with or without EGFRdel, but enhanced EGFR signaling in TC-1-EGFRdel cells (Figs. 5C and S4A). Notably, DUSP22 deletion did not affect PD-L2 expression in HCC827 or H1650 cells (Fig. S4B). These results indicate that DUSP22 regulates the PD-L1 expression in lung cancer cells via EGFR-dependent and independent pathways, beyond its role in EGFR suppression for cell growth. Next, we established clinical significance of DUSP22 relative to PD-L1 expression in the TCGA LUAD dataset. Decreased DUSP22 expression was associated with significantly higher PD-L1 levels in LUAD patients (Fig. 5D, p = 0.00405). No correlation was found between DUSP22 and PD-L2 in the same dataset (Fig. S4C). These findings suggest that DUSP22 expression may serve as a predictive marker for PD-L1 expression, reflecting the influence of EGFR-dependent or independent pathways modulated by DUSP22.

### DUSP22 suppresses PD-L1 expression via inhibition of EGFR/c-Met and their downstream signaling pathways

**Fig. 6:**
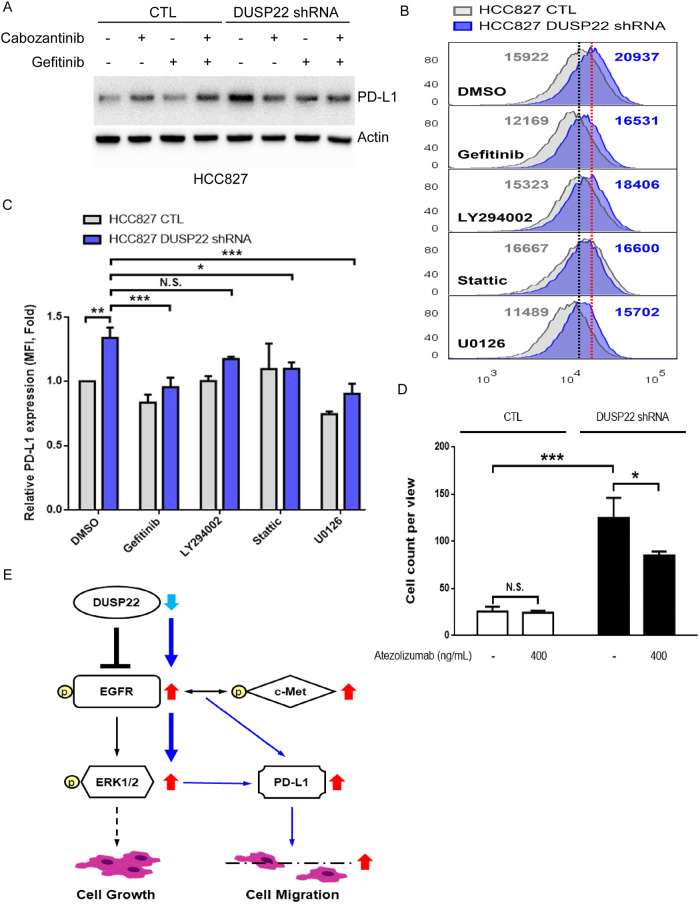
DUSP22 expression in tumor cells inhibits PD-L1 expression via EGFR, c-Met and its downstream signaling. **A** Treatment of gefitinib (100 nM) or cabozantinib (5 μM) inhibited PD-L1 upregulation in DUSP22-deleted HCC827 cells. **B**, **C** HCC827 cells were treated with gefitinib (100 nM), LY294002 (10 μM), Stattic (5 μM), or U0126 (20 μM). Quantification of flow cytometric analysis of surface PD-L1 protein expression. n = 3 per group. Data are representative of three independent experiments. The data are presented as the mean ± SD. *p < 0.05; **p < 0.01; ***p < 0.001. NS not significant. **D** Inhibition of DUSP22 deletion-induced cell migration by anti-PD-L1 antibody (Atezolizumab) treatment. The cell migration assay was carried out in the presence or absence of an anti-PD-L1 antibody. n = 3 per group. Data are representative of three independent experiments. The data are presented as the mean ± SD. Statistical analysis was determined by one-way ANONA for C-D. *p < 0.05; **p < 0.01; ***p < 0.001. **E** The working model of the EGFR/c-Met signaling repressed by DUSP22 for cell proliferation and migration in lung cancer cells.

To investigate the molecular link between DUSP22 deletion and increased PD-L1 expression in lung cancer cells, we found that PD-L1 levels decreased significantly in DUSP22-deleted HCC827 cells treated with gefitinib or cabozantinib alone (Fig. 6A), suggesting that the upregulation of PD-L1 due to DUSP22 deletion is mediated through EGFR and c-Met pathways. By treating DUSP22-deleted HCC827 cells with various inhibitors, we identified that gefitinib, Stattic, and U0126 effectively reduced PD-L1 levels induced by DUSP22 deletion, but LY294002 (PI3K/Akt inhibitor) did not (Fig. 6B, C). These findings underscore the crucial role of DUSP22 in negatively regulating the EGFR downstream pathway and suppressing PD-L1 expression. In contrast, inhibitors except for Sttatic were ineffective in reducing PD-L1 expression induced by DUSP22 deletion in gefitinib-resistant H1650 cells (Fig. S5A–C), suggesting that the activation of STAT3 due to DUSP22 deletion did not contribute to Akt-mediated resistance in H1650 cells. Additionally, DUSP22 deletion did not affect the sensitivity of these cells to gefitinib and cabozantinib. However, it did moderately suppress colony formation and proliferation in H1650 cells (Fig. S6A–C). These results indicate that PTEN deletion-induced gefitinib resistance in H1650 cells through sustained Akt activity may serve as a common resistance mechanism to inhibitors targeting EGFR downstream signaling for cell survival and gene expression [12]. More importantly, studies have shown that tumoral PD-L1 plays a role in controlling tumor cell motility for spread [38, 39]. To investigate the impact of PD-L1 neutralization on HCC827 cancer cell migration heightened by DUSP22 deletion, we performed a cell migration assay with and without an anti-PD-L1 antibody (Atezolizumab). The findings demonstrated that the anti-PD-L1 antibody effectively inhibited HCC827 cancer cell migration induced by DUSP22 deletion (Fig. 6D). Together, our results suggest that DUSP22 negatively regulates cell migration through EGFR/c-Met and PD-L1-dependent pathways.

## Discussion

Recent studies indicate that protein phosphatases play a crucial role in regulating kinase pathways in various cancer cells, functioning either as tumor suppressors or oncogenes by dephosphorylation processes [40]. For example, genetic changes affecting PTEN can drive lung tumorigenesis by activating mTOR/Akt pathways, leading to lung cancer metastasis [41]. Notably, PTEN is a significant prognostic marker for lung cancer patients, with loss of PTEN function linked to poorer outcomes [42]. Here, we investigated the role of DUSP22 in lung cancer development by studying its function in suppressing hyperactive EGFR signaling, a key oncogenic driver in LUAD [43]. Our study showed that DUSP22 acts as an EGFR phosphatase in lung cancer cells, inhibiting EGFR activity and its crosstalk with c-Met. This novel mechanism hinders crucial processes like cancer cell proliferation, migration, and PD-L1 expression. Additionally, studies have shown that PTEN loss results in sustained Akt and EGFR activation, providing a survival advantage and potential resistance to EGFR inhibitors in lung cancer cells [12, 44]. In contrast, DUSP22 deletion enhances HCC827 cell sensitivity to gefitinib, suggesting that DUSP22 loss promotes EGFR dependence in lung cancer, and targeting EGFR could be a more effective therapeutic approach for DUSP22-deficient lung tumors. Enforced DUSP22 expression significantly suppresses the growth of erlotinib-resistant H1650 lung cancer cell-based tumor by inhibiting EGFR and downstream ERK1/2 while not affecting sustained Akt activity. Targeting the EGFR/ERK1/2 axis through DUSP22 may combat PTEN deletion-induced EGFR TKI resistance. Our results support a combined EGFR and ERK1/2 targeting strategy for overcoming resistance in EGFR TKI-resistant lung cancer, as demonstrated in one recent study [45].

Unexpectedly, depleting DUSP22 revealed a toxicity mechanism driven by active ERK1/2, causing growth arrest in HCC827 and H1650 cells. This is consistent with one study on DUSP6, highlighting the importance of another DUSP enzymes in regulating ERK1/2 to prevent cell death induced by aberrant ERK1/2 activation in lung cancer [32]. These results challenge the idea that ERK1/2 activation due to DUSP downregulation drives treatment resistance and tumor progression. DUSP6 research emphasizes the need for tight control over the DUSP6/ERK1/2 axis to prevent hyperactivity-related toxicity and support tumor cell functions [32]. DUSP22 deletion in lung cancer cells increases dependence on EGFR activity for growth and survival, as it leads to co-activation of EGFR and ERK1/2. Proper regulation of ERK1/2 by DUSP enzymes is crucial to avoid detrimental consequences of unchecked ERK1/2 activity.

Previously, Li et al. showed that DUSP22 inhibits H1299 cell migration through FAK dephosphorylation [18]. However, the increase in FAK activity by EGFR or c-Met inhibitors does not align with the suppression of cell migration by both inhibitors, indicating FAK may not drive EGFR or c-Met-mediated migration [8]. Studies have indicated that gefitinib and cabozantinib activate FAK through various pathways, such as osteopontin induction and integrin signaling [46, 47]. Our study excludes FAK involvement in DUSP22 deletion-induced cell migration. The inhibition of cell migration and signaling induced by DUSP22 deletion by gefitinib and cabozantinib supports a working model where EGFR and c-Met RTKs exhibit mutual dependence following DUSP22 depletion. Consistently, treatment with gefitinib or cabozantinib markedly reduced PD-L1 expression in HCC827 cells with DUSP22 deletion, linking PD-L1 upregulation to increased cell migration in DUSP22-deleted lung cancer cells. Therefore, combining EGFR or c-Met TKIs with anti-PD1/PD-L1 immunotherapy for DUSP22-depleted EGFRdel-driven mouse lung tumors appears promising. However, several clinical trials have not shown significant benefits from PD-1/PD-L1 blockade alone or with an EGFR TKI in EGFR mutant lung cancer patients [48]. Further study is necessary to discover improved treatment strategies for EGFR mutant lung cancer patients with DUSP22 loss or downregulation. Here, we have developed a working model demonstrating that DUSP22 negatively regulates cell growth and migration in lung cancer cells through EGFR/c-Met and PD-L1-dependent pathways (Fig. 6E).

In summary, our study has provided insights into the tumor-intrinsic mechanisms of DUSP22 in suppressing lung cancer via EGFR. Our bioinformatics data suggest that LUAD patients with low DUSP22 expression had a worse prognosis, which may be potentially associated with increased EGFR/c-Met signaling, leading to further activation of downstream signaling and driving lung cancer cell growth, migration/invasion, and PD-L1 upregulation. Future translational studies are needed to test TKIs of EGFR/c-Met and PD-L1 blockade to inhibit lung cancer progression resulting from loss of DUSP22 function. Lastly, the mRNA-lipid nanoparticle (LNP) technology has emerged as the leading method for gene delivery, primarily due to the successful development of COVID-19 vaccines [49]. By utilizing synthetic mRNA encoding DUSP22 and coating it with LNP, it becomes possible to directly deliver DUSP22 to lung cancer cells that lack sufficient levels of this gene. This approach has the potential to harness DUSP22 expression, enabling the simultaneous targeting of EGFR and ERK1/2 pathways, resulting in the suppression of TKI-resistant or DUSP22-deleted tumor growth.

## Material and methods

### Cell culture and chemicals

A549, H1299, HCC827, H1650, H1975, TC-1, H520, LK2, and LL/2 cells (originally from ATCC) were obtained from the NHRI cell line bank, verified by cell line authentication using STR (short tandem repeat) DNA genotyping, and cultured in RPMI 1640 medium (#11875-093; Gibco, Thermo Fisher Scientific, Waltham, MA, USA) supplemented with 10% fetal calf serum (10437-028; Gibco, Thermo Fisher Scientific, Waltham, MA, USA), 2 mM L-Glutamine (#GLL01; Caisson Laboratories, Smithfield, UT, USA), and 1% penicillin/streptomycin (#15140-122; Gibco, Thermo Fisher Scientific, Waltham, MA, USA). Cell lines were routinely confirmed to be free of mycoplasma contamination using PCR analysis. For in vitro treatment, BML260 (#141733; Abcam, Cambridge, UK), gefitinib (#ab142052; Abcam, Cambridge, UK), LY294002 (#1130; Tocris Bioscience, Bristol, UK), Stattic (#S7024; Selleck Chemicals LLC, Houston, TX, USA), U0126 (#1144; Tocris Bioscience, Bristol, UK), and cabozantinib (#S1119; Selleck Chemicals LLC, Houston, TX, USA) were each dissolved in DMSO (#D2650; Sigma-Aldrich, Merck KGaA, Darmstadt, Germany) and stored at −2 °C; DMSO was used as vehicle control.

### Statistics analysis and reproducibility

The lung adenocarcinoma dataset from TCGA (https://cancergenome.nih.gov/) was analyzed with R/Bioconductor. Survival analysis of this dataset was performed using OncoLnc (http://www.oncolnc.org/) [30] and Kaplan-Meier plotter [50]. The log-rank test was used for Kaplan–Meier survival analyses. RNA sequencing data were retrieved and analyzed to identify genes positively and negatively correlated with DUSP22 expression. Identified genes were sent for functional enrichment analysis on The Database for Annotation, Visualization and Integrated Discovery (DAVID; https://david.ncifcrf.gov/) [51, 52]. The Cox proportional hazards regression model was used to perform univariate and multivariate survival analysis using R. The TNM staging system is used for staging LUAD patients: T (tumor) describes the size and extent of the primary tumor, and the number after T (TX-T4) refers to the tumor size and/or extent of the main tumor; N (lymph node) indicates whether cancer has spread to nearby lymph nodes, and the number after N (NX-N3) provides information about the number of affected lymph nodes; M (metastasis) indicates whether the cancer has metastasized or spread to distant organs or tissues beyond the primary site, and the number after M (MX-M1) determine the presence or absence of distant metastases. Stage I, II, III, and IV were used to classify the extent and progression of cancer. Each stage represents a different level of tumor growth, invasion, and spread. Prism v.7.0. software (GraphPad Software, San Diego, CA, USA) was used for graphs and statistical analyses. Each experiment in this study was performed more than three times with similar results, and the data presented in figures are representative of multiple independent experiments. Differences between two groups of data were determined using the Student’s *t* test or the Wilcoxon Rank Sum Test. For comparison of more than two groups, one-way or two-way analysis of variance (ANOVA) were used for statistical analysis. *p* values less than 0.05 were considered statistically significant. Not significant: n.s, *p < 0.05; **p < 0.01; ***p < 0.001.

A detailed description of the materials and methods used in this study is available in the online Supplementary Material.

### Supplementary information

Supplementary Figures 1-6

Supplementary Materials and Methods

Raw image file of all Western blot images

## Data Availability

Data sharing not applicable to this article as no datasets were generated or analyzed during the current study. All data generated or analyzed during this study are included in this published article and its supplementary figure files.
