# Supplementary figures and images for: DUSP22 inhibits lung tumorigenesis by suppression of EGFR/c-Met signaling
Source: Cell Death Discov. 2024 Jun 14;10:285. doi: 10.1038/s41420-024-02038-8 (PMC11178881; doi:10.1038/s41420-024-02038-8)

A

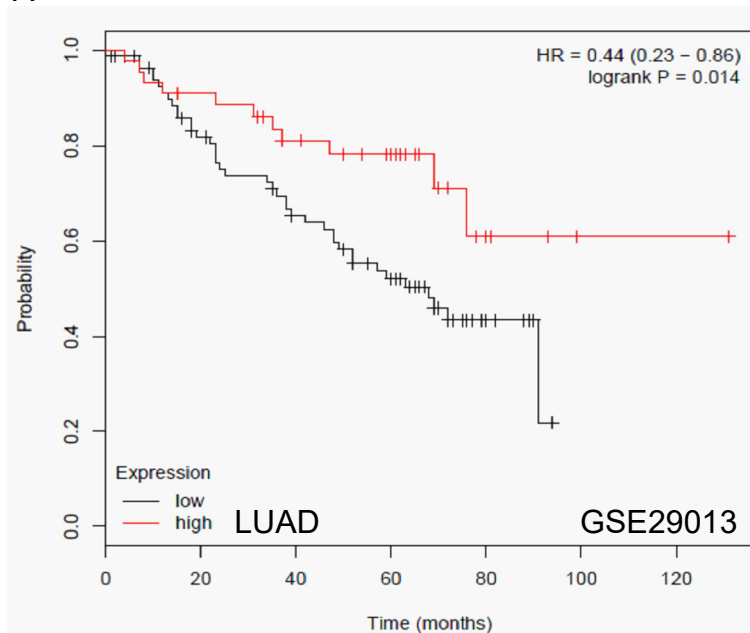

B

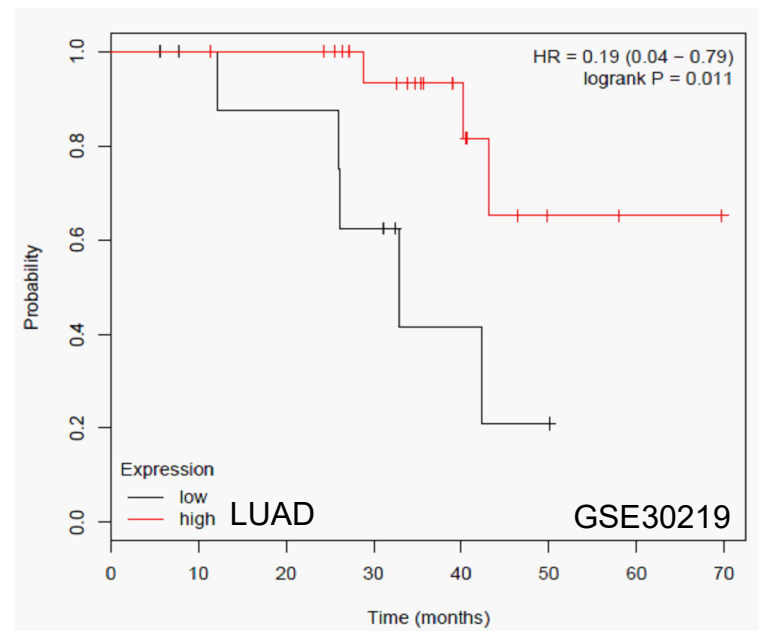

C

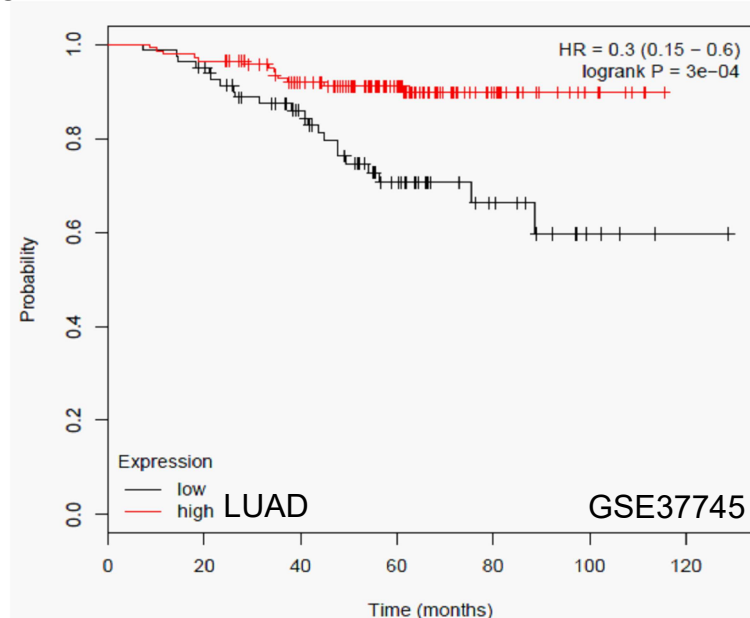

D

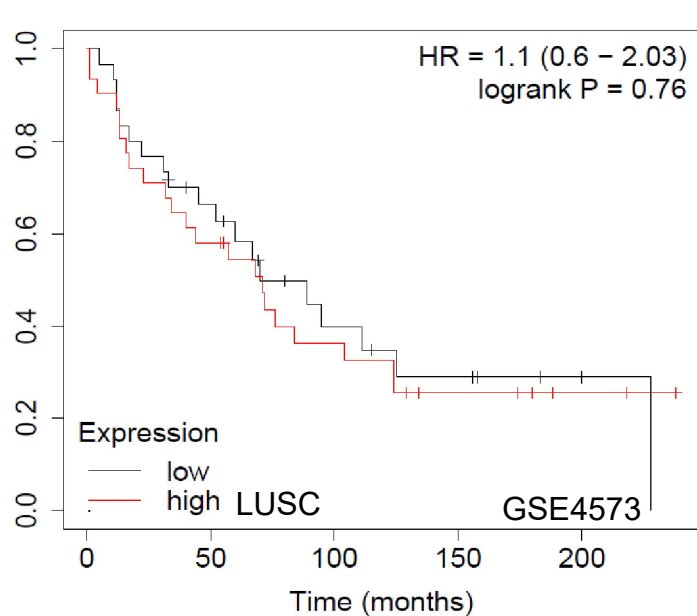

E

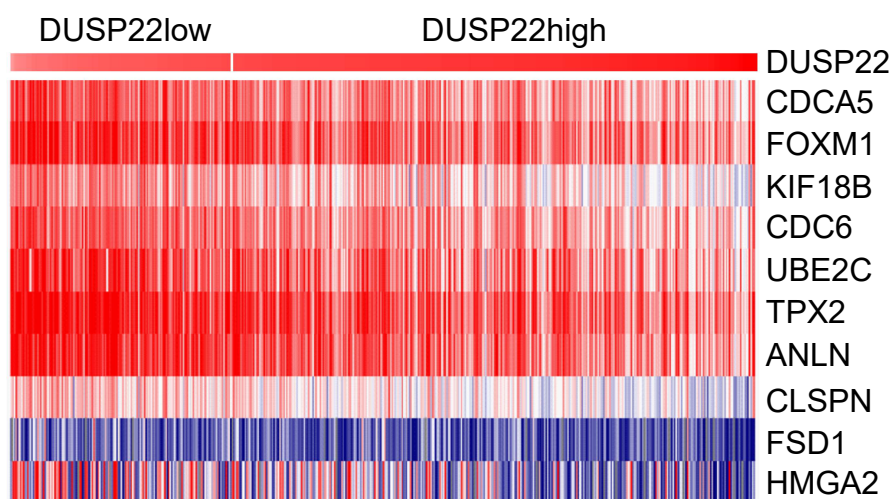

A

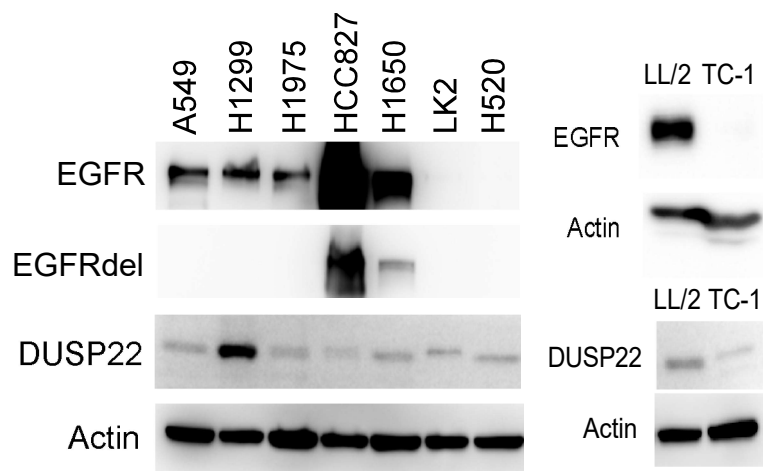

B

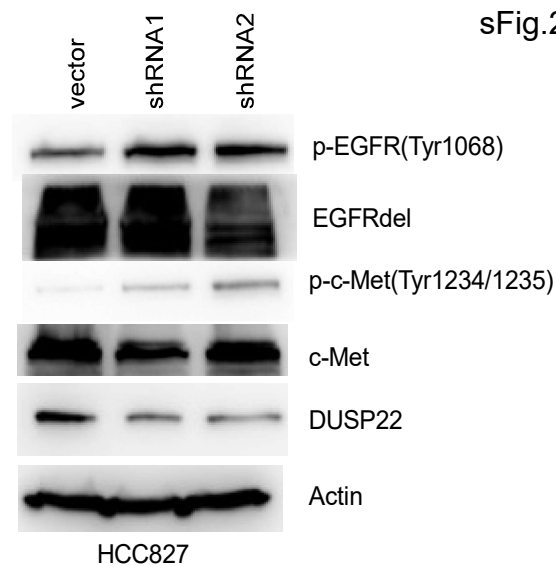

C

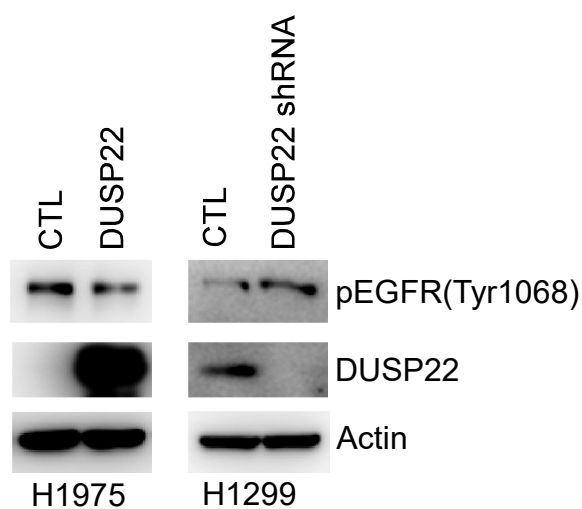

D

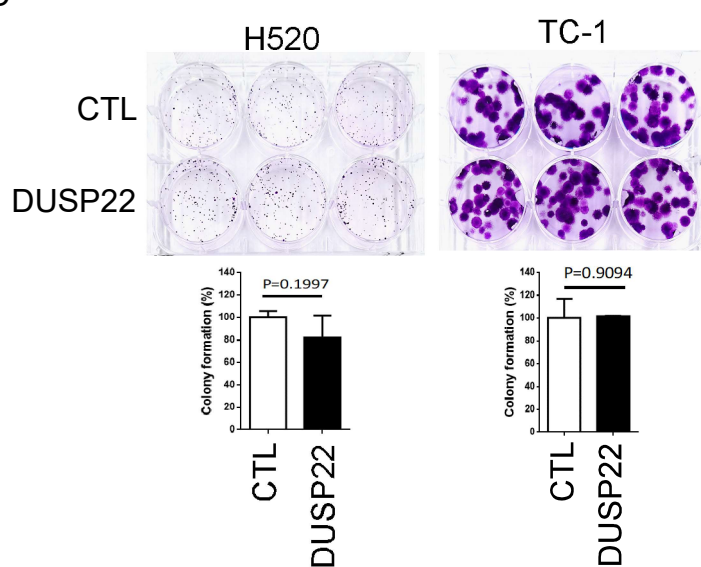

E

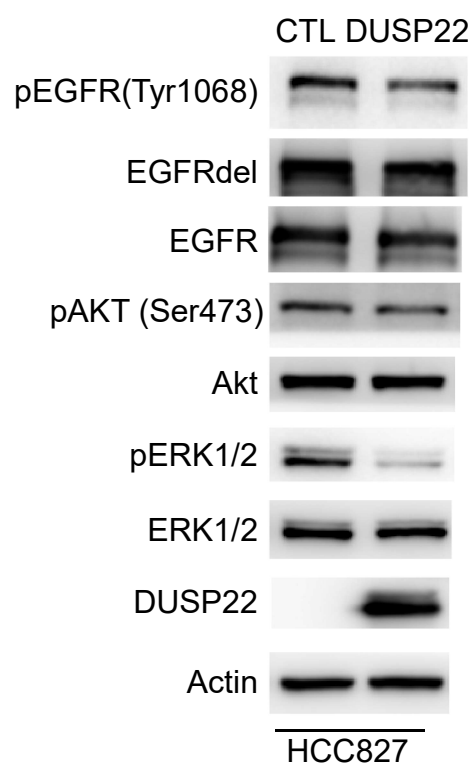

F

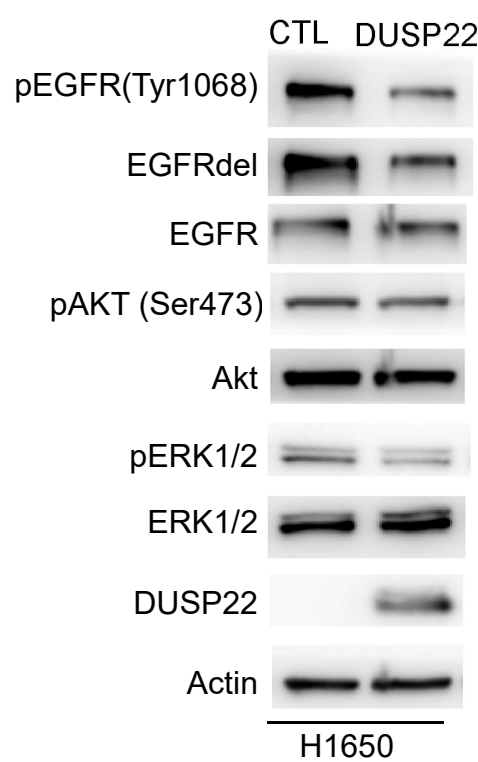

G

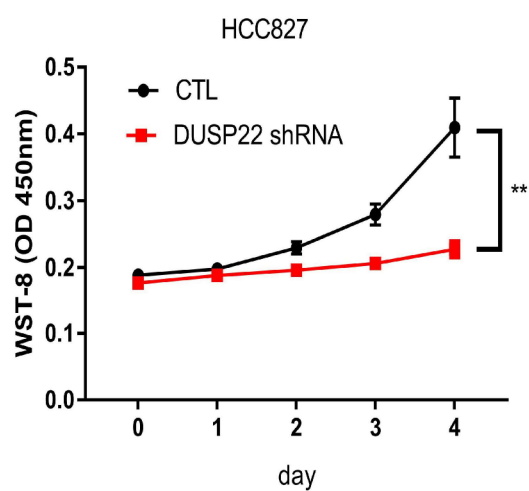

A

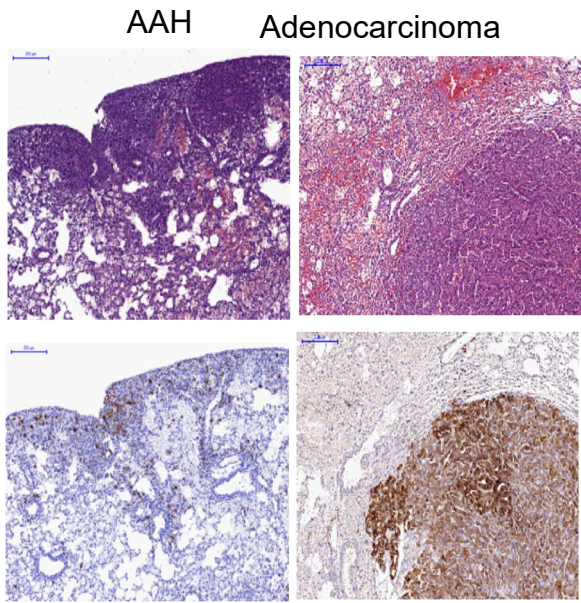

B

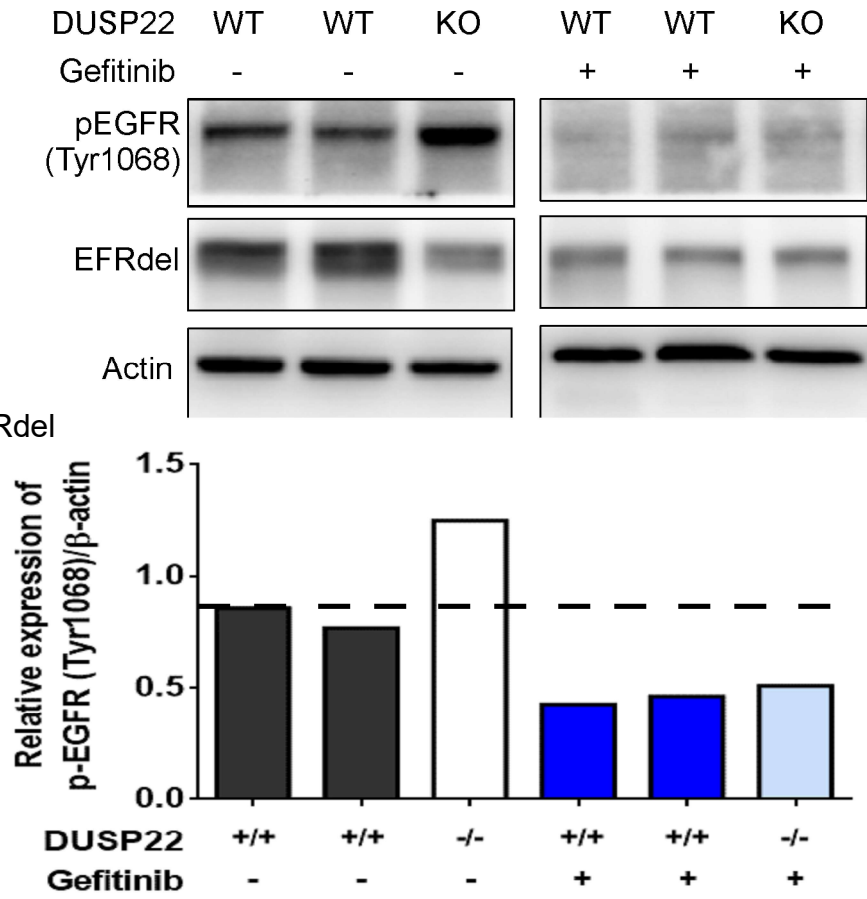

C

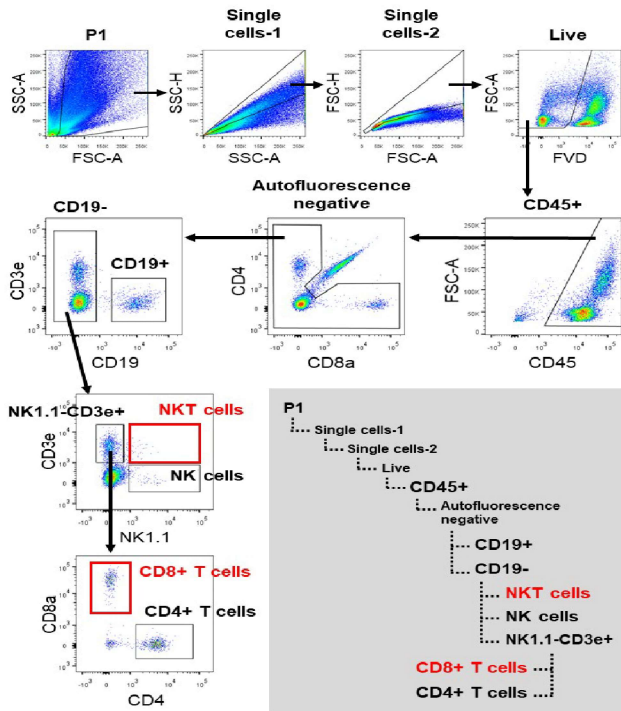

D

Cabozantinib ( $\mu$ M)

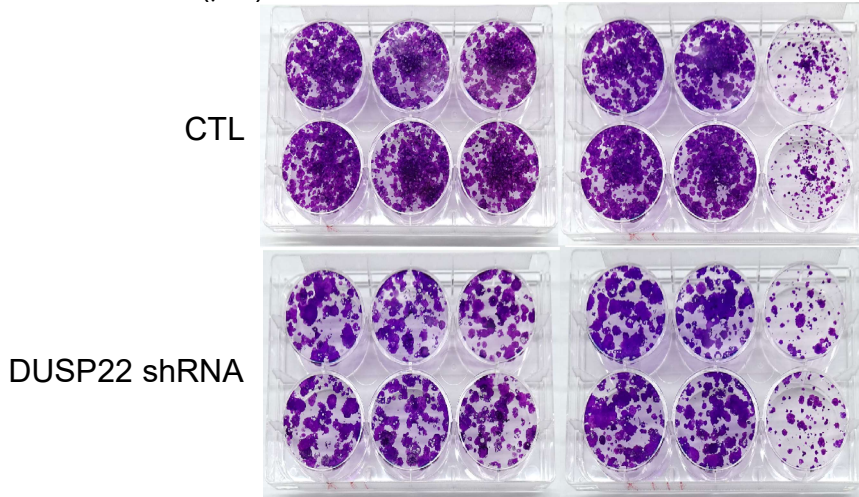

HCC827

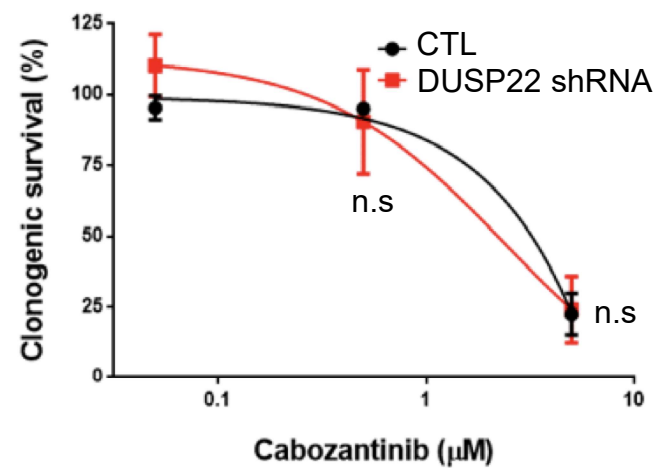

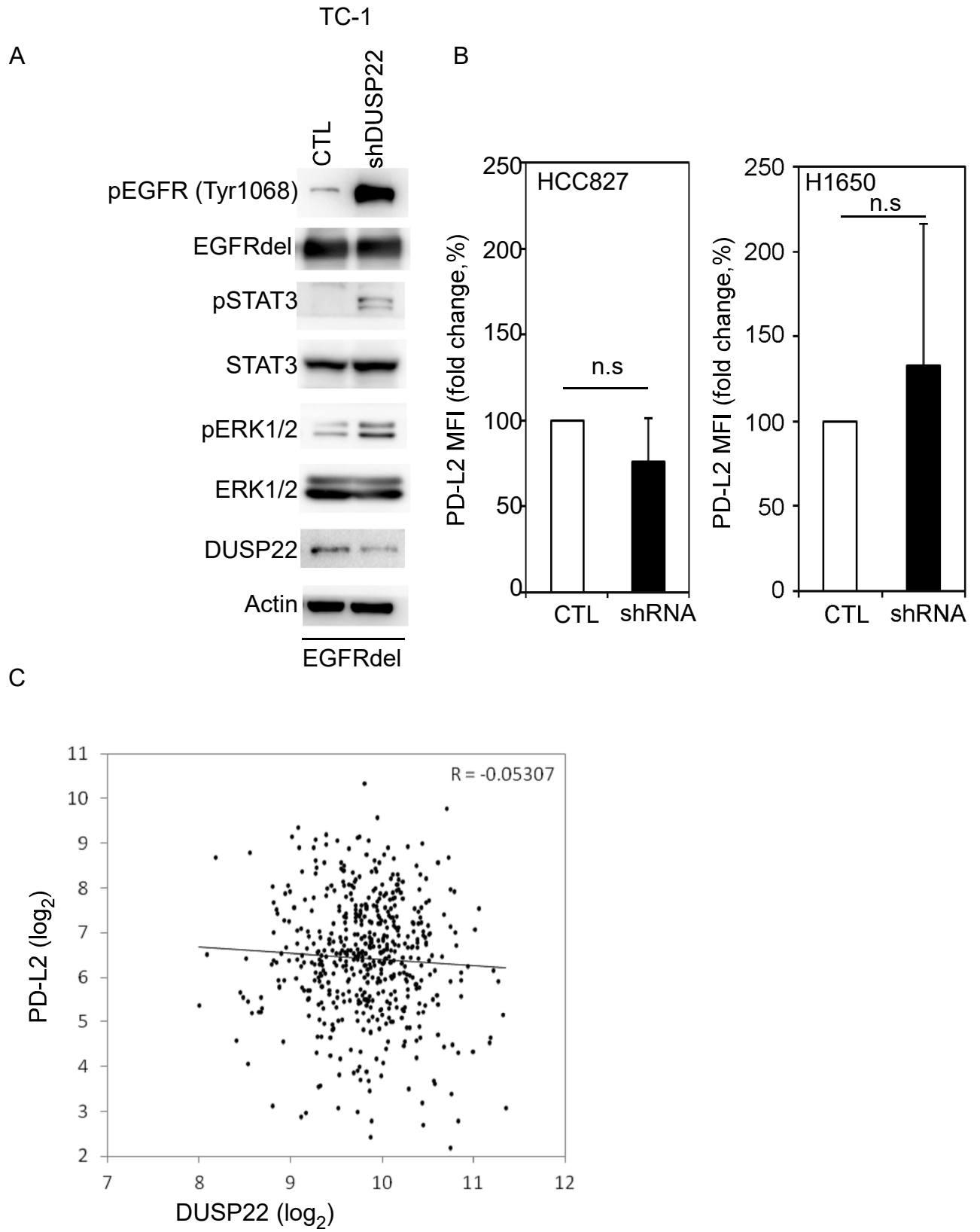

A

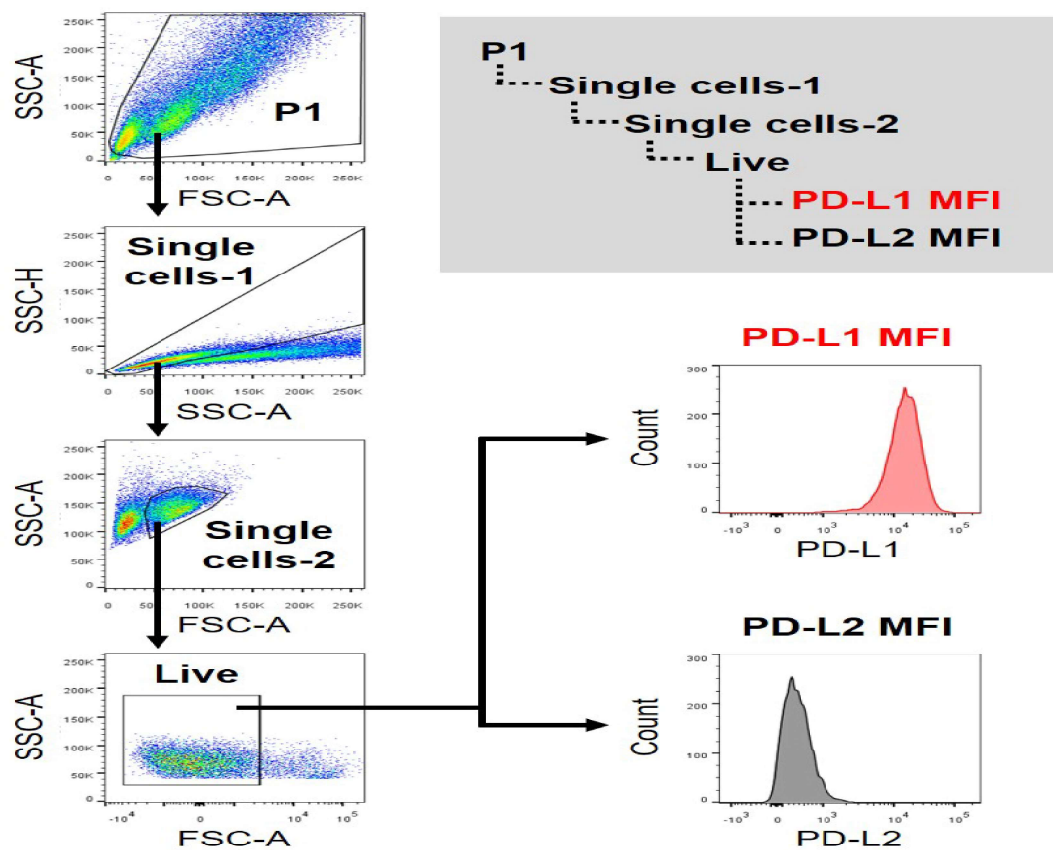

B

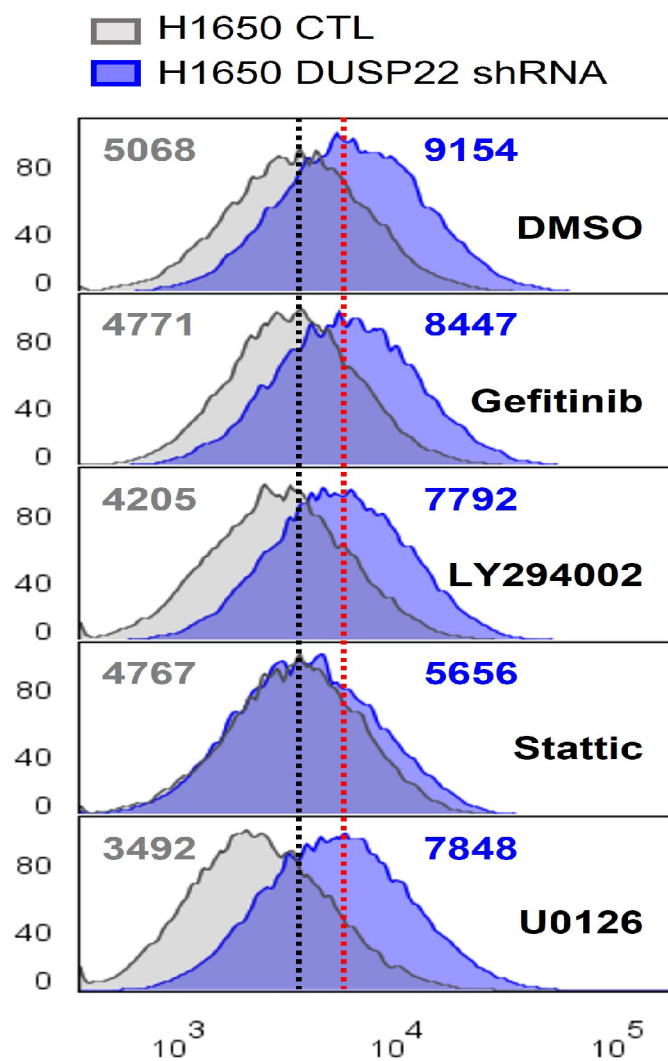

C

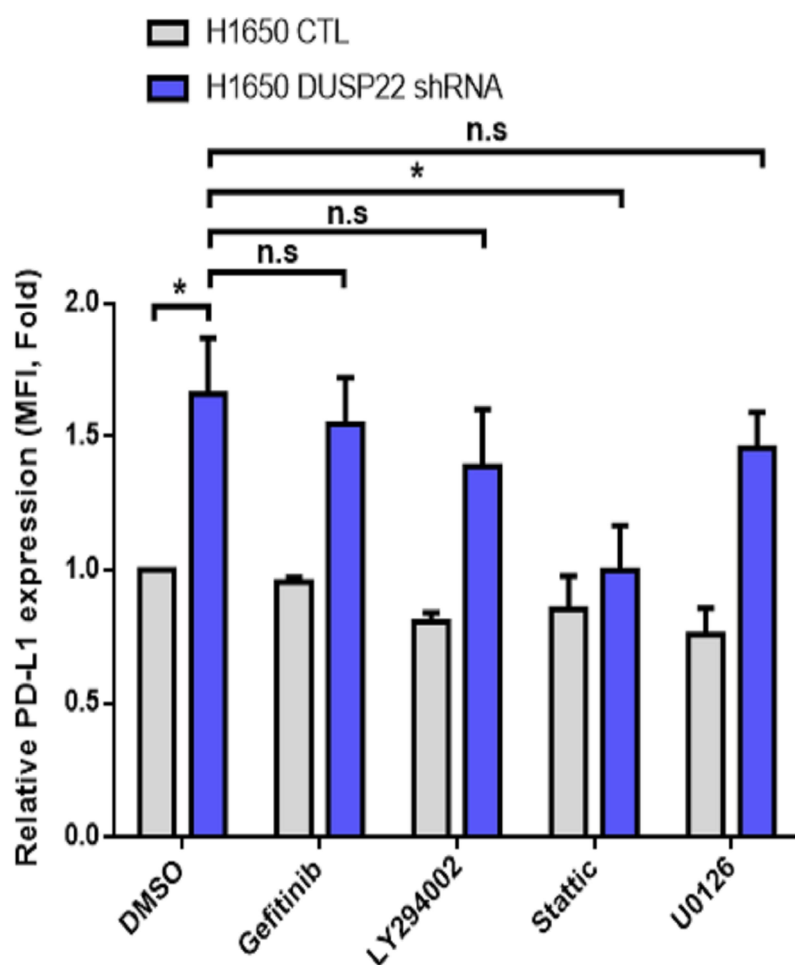

A

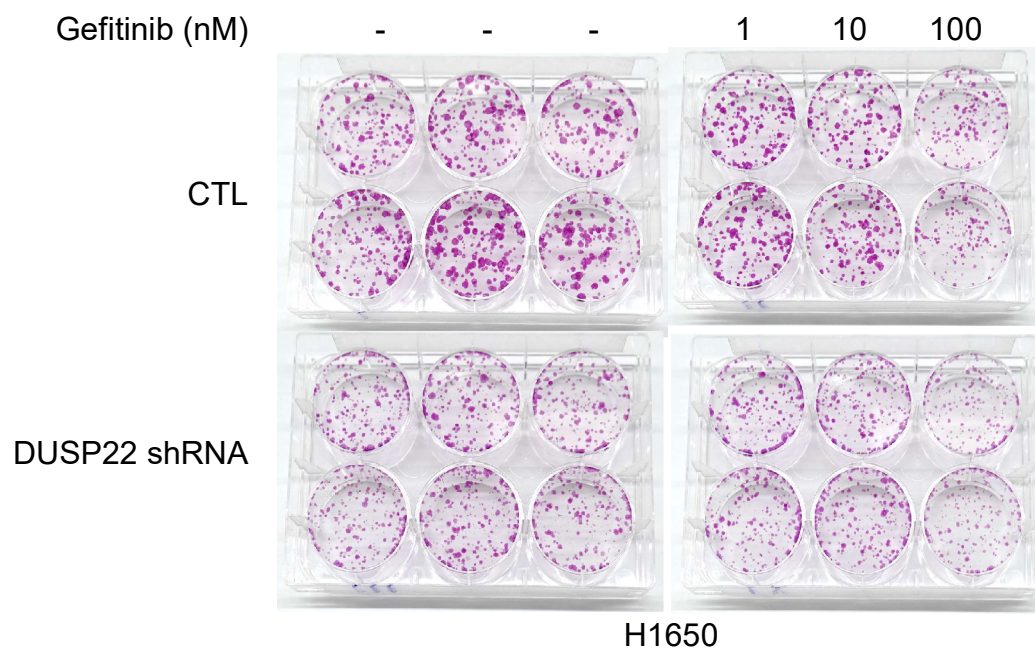

B

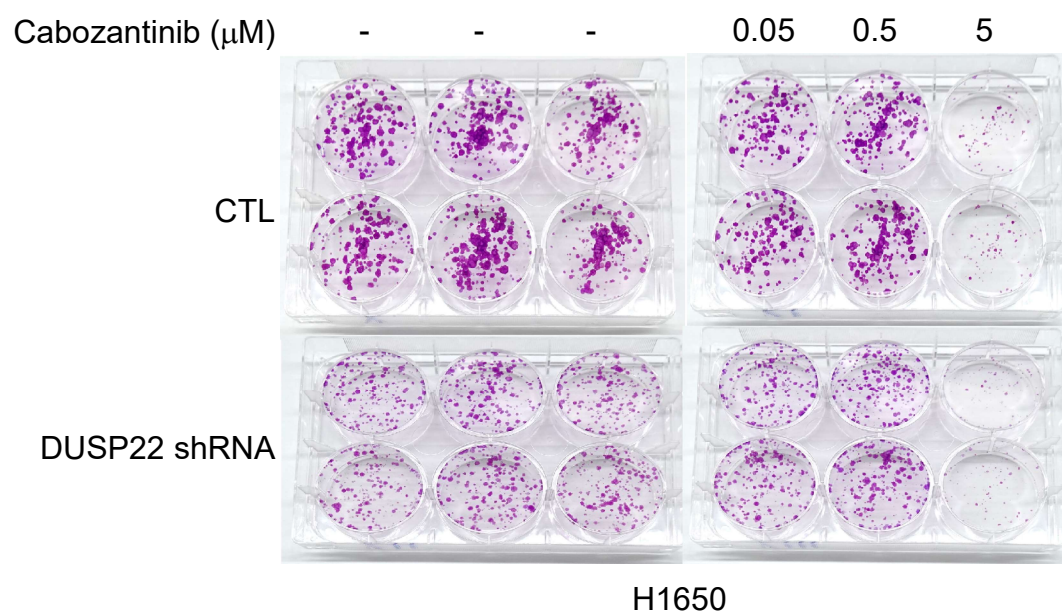

C

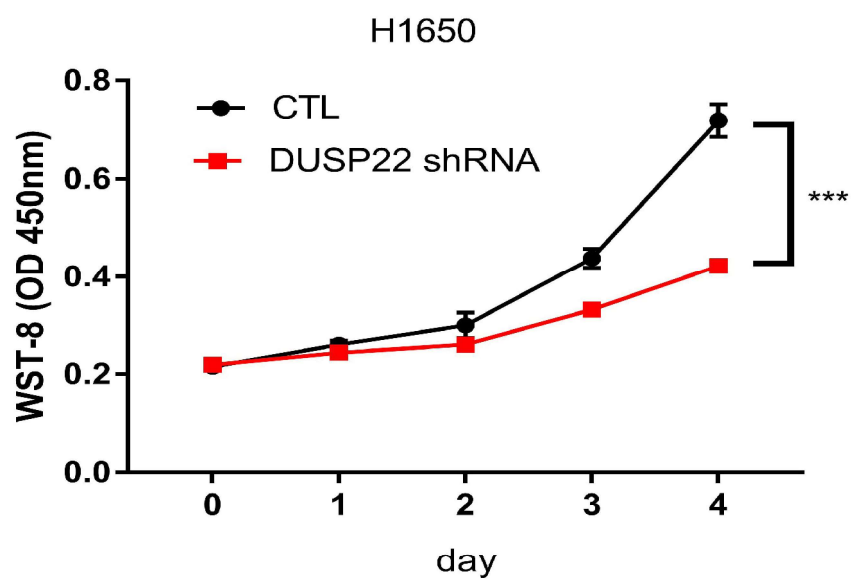

Supplement: Supplementary file 1 — Supplementary Figures 1-6 [file 41420_2024_2038_MOESM1_ESM.pdf]
