# Supplementary material for: DUSP22 inhibits lung tumorigenesis by suppression of EGFR/c-Met signaling
Source: Cell Death Discov. 2024 Jun 14;10:285. doi: 10.1038/s41420-024-02038-8 (PMC11178881; doi:10.1038/s41420-024-02038-8)
Supplement: Supplementary file 2 — Supplementary Materials and Methods [file 41420_2024_2038_MOESM2_ESM.docx]

**Supplementary file for**

**Dual-specificity phosphatase 22 (DUSP22) functions as a tumor suppressor by targeting EGFR signaling in lung cancer**

Hsiao-Han Lin^1,#^, Cheng-Wei Chang^1,#^, Yu-Ting Liao^1^, Shauh-Der Yeh^2^, Hsiu-Ping Lin^1^, Hui-Min Ho^1^, Chantal Hoi-Yin Cheung^3^, Hsueh-Fen Juan^3^, Yi-Rong Chen^4^, Yu-Wen Su^1^, Li-Mei Chen^1^, Tse-Hua Tan^1^, and Wen-Jye Lin^1,*^

^1^Immunology Research Center, National Health Research Institutes, Miaoli County, 35053 Taiwan

^2^Department of Urology, Graduate Institute of Clinical Medicine, Taipei Medical University, Taipei, Taiwan

^3^Department of Life Science, National Taiwan University, Taipei, 10617, Taiwan

^4^Institute of Molecular and Genomic Medicine, National Health Research Institutes, Miaoli County, 35053, Taiwan

# Contributed equally

**Correspondence**: Wen-Jye Lin, PhD, Immunology Research Center, National Health Research Institutes, Zhunan, Miaoli County, Taiwan 35053, Telephone: +886-37-246166 ext.37605, Fax: +886-37-586642, E-mail: 020808@nhri.edu.tw

**Running Title**

DUSP22 regulates the EGFR and c-Met axis

**Keywords:** DUSP22, EGFR, c-Met, lung cancer, Dual-specificity phosphatases

Conflict of interest: The authors declare no conflict of interest.

**Supplementary Material and Methods**

**Colony Formation Assay**

For the colony formation assay, cells were seeded in 6-well low attachment plates at a density of 200-500 cells per well in presence or absence of gefitinib or cabozantinib. After 14 days, the tumor colonies were stained with 1% crystal violet (#32675; Sigma-Aldrich, Merck KGaA, Darmstadt, Germany) in 30% ethanol, washed with water, and air dried. Colonies were counted manually using a microscope.

**DUSP22 overexpression and knockdown**

DUSP22 overexpression and shRNA knockdown lentiviral plasmids were constructed using a pLAS2.1w.PeGFP-I2-Puro vector and a pLKO_AS1010 vector, respectively, which were purchased from the RNA Technology Platform and Gene Manipulation Core, Institute of Molecular Biology, Academia Sinica, Nankang, Taipei, Taiwan. Lentiviral production followed the protocol of the RNA Technology Platform and Gene Manipulation Core. Stable DUSP22 overexpression and shRNA knockdown clones were established through lentivirus infection for 24 hr and subsequent puromycin selection; selected cells were maintained in growth medium containing 2 μg/ml puromycin (#A1113803; Gibco, Thermo Fisher Scientific, Waltham, MA, USA). Overexpression and knockdown efficiencies were verified with real-time PCR and Western blotting.

**Mouse experiments**

DUSP22 KO mice and EGFR-del (exon 19 deletion, ∆746-750) transgenic mice over-expressing the EGFR-del mutation, under the control of a surfactant protein C (SPC) promoter were established as described previously [1-2]. First, DUSP22 KO mice (C57BL/6 background) were bred with EGFR-del transgenic mice (C57BL/6 background) in-house to generate EGFR-del/DUSP22 heterozygous mice. Then EGFR-del/DUSP22 heterozygous mice were intercrossed to generate EGFR-del/DUSP22 KO mice. Mice, both male and female, with EGFR-del/DUSP22 WT or EGFR-del/DUSP22 KO genotypes, were sacrificed beginning at 9 months for the following experiments. Mice were euthanized, and whole lung tissues were collected after whole blood collection by cardiac puncture. Small pieces of each lobe of the lung were collected and fixed in 10% formaldehyde for histology and immunohistochemistry (IHC). Half of the left lung was snap frozen in liquid nitrogen and stored at -80 °C for subsequent RNA or protein extraction. Other parts of the lungs were processed for flow cytometric analysis. Gefitinib (#S1025; Selleck Chemicals LLC, Houston, TX, USA) was solubilized in a solution of 1% Tween 80 (#P6474; Sigma-Aldrich, Merck KGaA, Darmstadt, Germany) with H_2_O. Mice aged 32 to 36 weeks were administered a dose of 100 mg/kg intraperitoneally three times per week for two weeks, alongside a control receiving the vehicle alone. Lung tissues were harvested for protein extraction and subsequent Western blot analysis. All experimental protocols were performed in accordance with the guidelines for animal experimental research of the National Health Research Institutes (NHRI), which were approved by the Institutional Animal Care and Use Committees of NHRI.

**Immunohistochemistry**

IHC staining was performed on 4-μm-thick, formalin-fixed, paraffin-embedded mouse lung samples. Slides were incubated with a primary antibody (1:100 dilution) against EGFR-del (#2085; Cell Signaling Technology, Danvers, MA, USA) overnight at room temperature. Detailed procedures were as previously described [3].

**Lung pathology of EGFR-del/DUSP22 WT and KO mice**

Hematoxylin and eosin (H&E) staining of 10% formaldehyde-fixed paraffin-embedded lung sections and IHC of lung tissue sections from EGFRdel (WT vs. DUSP22 KO) mice were examined for alveolar adenomatous hyperplasia (AAH) and adenoma or adenocarcinoma by pathologists at the NHRI core facility.

**Flow cytometry and cytokine array**

Cells were collected and washed twice with PBS, and then stained with antibodies and viability dye in staining buffer (PBS containing 2% FBS and 0.1% sodium azide). Stained cells were washed twice with staining buffer, and then analyzed on a BD FACSCanto II flow cytometer. Results were analyzed using FlowJo software (BD Bioscience, Becton, Dickinson and Company, Ashland, OR, USA). Antibodies and dye were purchased from BioLegend (San Diego, CA, USA), BD Bioscience (Becton, Dickinson and Company, Franklin Lakes, NJ, USA) and eBioscience (Thermo Fisher Scientific, Waltham, MA, USA), including eFluor 506 anti-mouse CD45 (#69-0451, clone 30-F11), PerCP anti-mouse CD3e (#100326, clone 145-2C11), APC anti-mouse CD19 (#115512, clone 6D5), FITC-anti mouse CD4 (#100406, clone GK1.5), PE anti-mouse CD8a (#553033, clone 53-6.7), BV421 anti-mouse NK1.1 (#108732, clone PK136), APC anti-human PD-L1 (#329707, clone 29E.2A3), BV421 anti-mouse PD-L1 (#124315, clone 10F.9G2), BV421 anti-human PD-L2 (#329615, clone 24F.10C12), APC anti-mouse PD-L2 (#107210, clone TY25) and fixable viability dye (#65-0865). Cytokine levels were analyzed using the Bio-Plex Mouse Cytokine Group I 23-plex assay kit (Bio-Rad Laboratories) following the manufacturer's protocol. Tumor lysates were incubated with antibody-coupled beads, biotinylated detection antibodies, and streptavidin-PE before analysis on the Bio-Plex system with data processed using Bio-Plex Manager software version 6.0.

**RT-PCR**

Total RNA was isolated from cells or mouse tissues using the RNeasy Mini Kit (74104; Qiagen, Germantown, MD, USA), and converted to first-strand cDNA using SuperScript® III Reverse Transcriptase (#18080044; Invitrogen, Thermo Fisher Scientific, Waltham, MA, USA) and random primers according to the manufacturer's instructions. Gene expression analyses were performed via quantitative real-time PCR using the Roche LightCycler® PCR 480 system.

**Western blotting and phospho-RTK array**

Cells or frozen tissues were washed with cold 1x PBS and lysed in NP-40 Cell Lysis Buffer (#FNN0021; Invitrogen, Thermo Fisher Scientific, Waltham, MA, USA), with protease and phosphatase inhibitors, on ice for 30 min. Suspended cell lysates were centrifuged at 15,000 rpm for 15 min at 4 °C. The protein concentration of cell lysates was quantified using the Pierce BCA Protein Assay Kit (#23227; Thermo Fisher Scientific, Waltham, MA, USA). Equal amounts of lysates were subjected to SDS-PAGE electrophoresis and immunoblotting as described previously [4]. Antibodies used for this study include EGFR (#4267, 1:1000), EGFR (E746-A750 del, #2085, 1:500), phosphorylated EGFR (Y1068, #3777, 1:1000), ERK1/2 (#4695, 1:500), phosphorylated ERK (T202/Y204, #4370, 1:250), STAT3 (#9139, 1:1000), phosphorylated STAT3 (Y705, #ab76315, 1:500; Abcam, Cambridge, UK), DUSP22 (#16514-1-AP, 1:250; Proteintech, Rosemont, IL, USA) for cell lines, DUSP22 (1:250; immunized rabbit serum isolated by GeneTex, Irvine, CA, USA), c-Met (#8198, 1:1000), phosphorylated c-Met (Y1234/1235, #3077, 1:1000), FAK (#3285, 1:500), phosphorylated FAK (Y397, #3283, 1:250), AKT (#4691, 1:500), phosphorylated AKT (S473, #4060, 1:500), PD-L1 (#13684, 1:1000), and β-Actin (#A5441, 1:5000; Sigma-Aldrich, Merck KGaA, Darmstadt, Germany). If not specified, antibodies were obtained from Cell Signaling Technology (Danvers, MA, USA). For phospho-RTK antibody arrays (#ARY001B, R&D Systems, Minneapolis, MN, USA), cell lysates, with or without inhibitor treatment, were subjected to Western blot-based array according to the manufacturer’s instructions.

**Transwell migration assay**

To test cell migration, 1 × 10^5^ HCC827 cancer cells were suspended in 250 μl of medium without FBS and seeded in transwell insert chambers with an 8 μm porous PET membrane (#353097; Corning, NY, USA); bottom chambers contained 500 μl of medium containing 10% FBS. The HCC827 cells were treated with either a vehicle, a TKI, or Atezolizumab (an anti-PD-L1 antibody). Atezolizumab was purchased from Selleckchem (#A2004, USA), while purified human IgG1 Isotype from Biolegend (#403502, San Diego, California, USA) was employed as a control antibody for the cell migration assay. HCC827 cells were incubated under 5% CO_2_ at 37 °C for 18 hr; non-migrated cells in the tr answells were removed by suction and wet cotton swabs. Cells that migrated were stabilized with 100% methanol for 10 min at −20 °C, stained with 10% Giemsa, visualized using a phase-contrast microscope, and quantified using Image J software.

**Cell proliferation assay**

The cancer cells were plated in 96-well plates. WST-8 reagents (#96992; Sigma-Aldrich, Merck KGaA, Darmstadt, Germany) were added at each time point following the manufacturer’s protocol. The absorbance at 450 nm was then quantified using a microplate reader.

**References**

1 Li JP, Yang CY, Chuang HC, Lan JL, Chen DY, Chen YM *et al*. The phosphatase JKAP/DUSP22 inhibits T-cell receptor signalling and autoimmunity by inactivating Lck. Nat Commun 2014; 5: 3618.

2 Yang CH, Chou HC, Fu YN, Yeh CL, Cheng HW, Chang IC *et al*. EGFR over-expression in non-small cell lung cancers harboring EGFR mutations is associated with marked down-regulation of CD82. Biochim Biophys Acta 2015; 1852: 1540-9.

3 Wu YC, Chang IC, Wang CL, Chen TD, Chen YT, Liu HP *et al*. Comparison of IHC, FISH and RT-PCR methods for detection of ALK rearrangements in 312 non-small cell lung cancer patients in Taiwan. PLoS One 2013; 8: e70839.

4 Izumi K, Mizokami A, Lin HP, Ho HM, Iwamoto H, Maolake A *et al*. Serum chemokine (CC motif) ligand 2 level as a diagnostic, predictive, and prognostic biomarker for prostate cancer. Oncotarget 2016; 7: 8389-98.

**Supplementary Figure Legends**

**Figure S1. High DUSP22 expression is associated with better survival among patients with LUAD.**

For each LUAD Gene Expression Signature (GSE) dataset, LUAD patient data was divided into high and low DUSP22-expressing subgroups and then subjected to Kaplan-Meier survival analysis and the log-rank test. Kaplan-Meier survival curves for LUAD or LUSC patients with high and low DUSP22-expression are shown for the following datasets: (A) GSE29013, (B) GSE30219, (C) GSE37745, and (D) GSE4573. (E) Gene correlation analysis of the TCGA LUAD dataset shows that expression of cell cycle genes is negatively correlated with DUSP22 expression.

**Figure S2. Inhibition of EGFR signaling by DUSP22.**

(A) Western blot analysis of DUSP22 expression in different human and murine lung cancer cells. Total cell lysates (25 μg) from each cancer cell line were resolved by SDS-PAGE followed by immunoblotting with an anti-DUSP22 antibody (left panel: human, right panel: mouse). Actin was used as a loading control. (B) Western blot analysis of EGFRdel, c-Met, and their phosphorylated forms in HCC827 cells with two-independent shRNAs against DUSP22. Actin was used as a loading control. (C) Overexpression (left panel) or downregulation (right panel) of DUSP22 by lentiviral DUSP22 expression vectors or lentiviral DUSP22 shRNA vectors, respectively, resulted in a respective decrease or increase in p-EGFR (Tyr0168) levels in H1975 or H1299 lung cancer cells. (D) DUSP22 failed to inhibit cell proliferation in H520 and TC-1 cells. Colony-forming assays were performed to assess the proliferation of H520 and TC-1 lung cancer cells with or without DUSP22 expression. The number of colonies was counted, and images were captured. All experiments were conducted independently three times, and the data are presented as the mean ± SD. (E) Left panel: DUSP22 overexpression represses EGFR signaling in HCC827 cells. (F) Right panel: DUSP22 silencing by DUSP22 shRNA increases EGFR signaling in H1650 cells. (G) The growth of HCC827 cancer cells, both control and those with DUSP22 knockdown, was assessed using a WST-8 assay over a period of 96 hours. The data are presented as the mean ± SD. Statistical significance of tumor cell growth was determined using two-way ANOVA. **, p < 0.01.

**Figure S3. Gefitinib treatment inhibits EGFR activity in lung tissue from EGFRdel/WT and DUSP22 KO mice.** (A) Representative images of lung tissues obtained from mice with EGFRdel/DUSP22 WT genotype. The lung tumor sections were examined with H&E staining (upper panel) and were also subjected to IHC staining using an EGFRdel antibody (lower panel). AAH: atypical adenomatous hyperplasia. (B) EGFR and p-EGFR levels in lung tissues from 32-36 week-old EGFRdel/WT (wild-type; +/+), HET (heterozygous; +/-), and DUSP22 KO (knockout; -/-) mice were analyzed by Western blot after gefitinib treatments for 2 weeks (upper panel); expression level quantification of the indicated proteins from Western blots using Image J software (lower panel). (C) Gating strategy for immune cell analysis in Fig. 3D and 3E is shown. (D)The HCC827 cells were treated with increased concentrations of cabozantinib. The cell growth was assessed by the colony formation assay. The representative images (left panel) and survival fraction (right panel) of HCC827 cells (± DUSP22 shRNA) after 14 days of treatment with cabozantinib were show as indicated. The data are presented as the mean ± SD. Statistical significance of tumor cell growth was determined using two-way ANOVA. n.s: not significance.

**Figure S4. Inactivation of DUSP22 function by shRNA enhances EGFR signaling in EGFR^low^ TC-1 cells with exogenous human EGFRdel expression.** (A) DUSP22 depletion increased phosphorylated forms of EGFR, ERK1/2, and STAT3 in EGFR^low^ TC-1 cells with human EGFRdel expression. Lysates from TC-1 cells expressing human EGFRdel (± murine DUSP22 shRNA) were immunoblotted with specific antibodies, as indicated (right panel; CTL, control cells without DUSP22 shRNA). Parental LL/2 and TC-1 mouse tumor cells were used as controls for endogenous EGFR expression (left panel). (B) Surface PD-L2 protein in control and DUSP22-silenced HCC827 and H1650 cells was measured by flow cytometry. The data are presented as the mean ± SD. Unpaired t test was used to for statistical analysis. n.s, not significant. (C) Gene correlation analysis of the TCGA LUAD dataset showed that expression of PD-L2 does not correlate with low DUSP22 expression.

**Figure S5. Inhibitors failed to block PD-L1 upregulation by DUSP22 deletion except for stattic in gefitinib-resistant H1650 Cells.** (A) Gating strategy for PD-L1 analysis in Fig. 5B, 5C, 6B, 6C, S4B, and S5 is shown. (B) H1650 cells (± DUSP22 shRNA) were treated with gefitinib (100 nM), LY294002 (10 μM), Stattic (5 μM), or U0126 (20 μM). Surface PD-L1 levels were measured by flow cytometry and representative histograms are shown. (C) Quantitation results of FACS analysis for PD-L1 levels. Statistical analysis of PD-L1 expression data in H1650 cells. The data are presented as the mean ± SD. One-way ANOVA was used for statistical analysis. n.s, not significant; *: *P* < 0.05.

**Figure S6. Representative images of colony formation in H1650 cells (± DUSP22 shRNA) following 14-day treatments with gefitinib or cabozantinib as indicated.** (A, B) Colony-forming assays were carried out to determine the proliferation of H1650 (± DUSP22 shRNA) treated with gefitinib or cabozantinib. (C) The growth of H1650 cancer cells with or without DUSP22 shRNA was evaluated using a WST-8 assay over a 96-hour period. The data are presented as the mean ± SD. Statistical significance of tumor cell growth was determined using two-way ANOVA. ***, p < 0.001.
