# Supplementary material for: DUSP22 inhibits lung tumorigenesis by suppression of EGFR/c-Met signaling
Source: Cell Death Discov. 2024 Jun 14;10:285. doi: 10.1038/s41420-024-02038-8 (PMC11178881; doi:10.1038/s41420-024-02038-8)

Fig. 3C

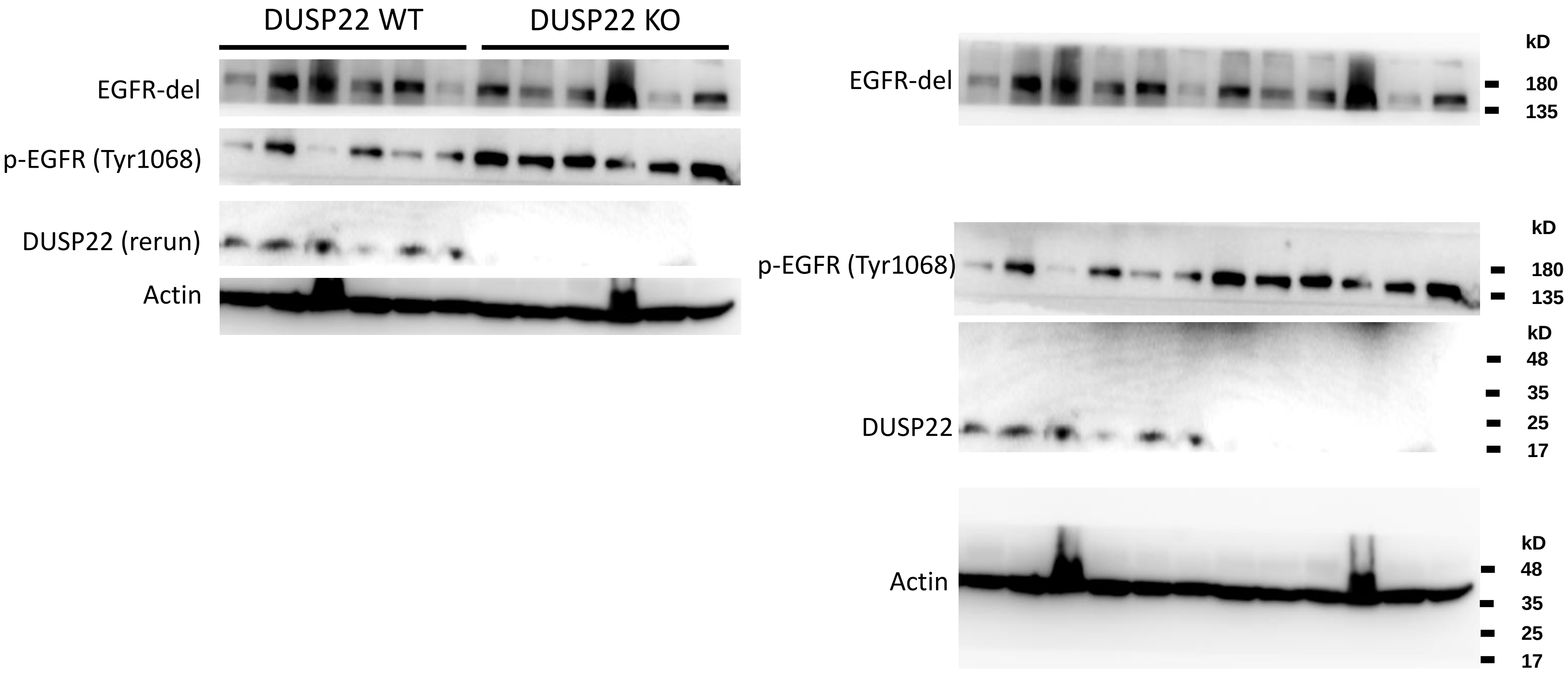
DUSP22 WT
DUSP22 KO
EGFR-del
p-EGFR (Tyr1068)
DUSP22 (rerun)
Actin
EGFR-del
kD
180
135
p-EGFR (Tyr1068)
kD
180
135
DUSP22
kD
48
35
25
17
Actin
kD
48
35
25
17

Fig. 4B

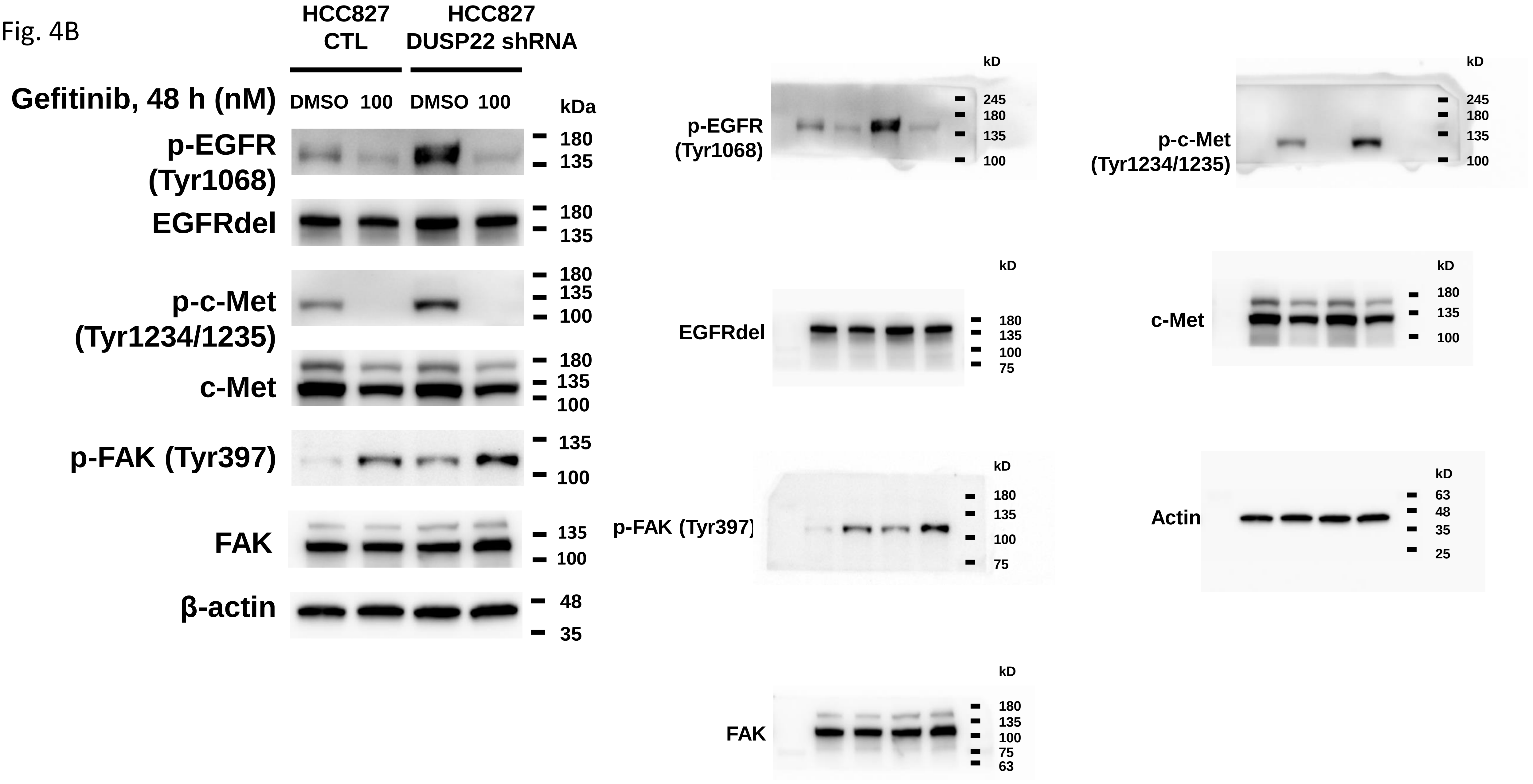

HCC827 CTL
HCC827 DUSP22 shRNA
Gefitinib, 48 h (nM)
DMSO
100
DMSO
100
kDa
p-EGFR (Tyr1068)
180
135
EGFRdel
180
135
p-c-Met (Tyr1234/1235)
180
135
100
c-Met
180
135
100
p-FAK (Tyr397)
135
100
FAK
135
100
β-actin
48
35
p-EGFR (Tyr1068)
kD
245
180
135
100
p-c-Met (Tyr1234/1235)
kD
245
180
135
100
EGFRdel
kD
180
135
100
75
c-Met
kD
180
135
100
p-FAK (Tyr397)
kD
180
135
100
75
Actin
kD
63
48
35
25
FAK
kD
180
135
100
75
63

Fig. 4D

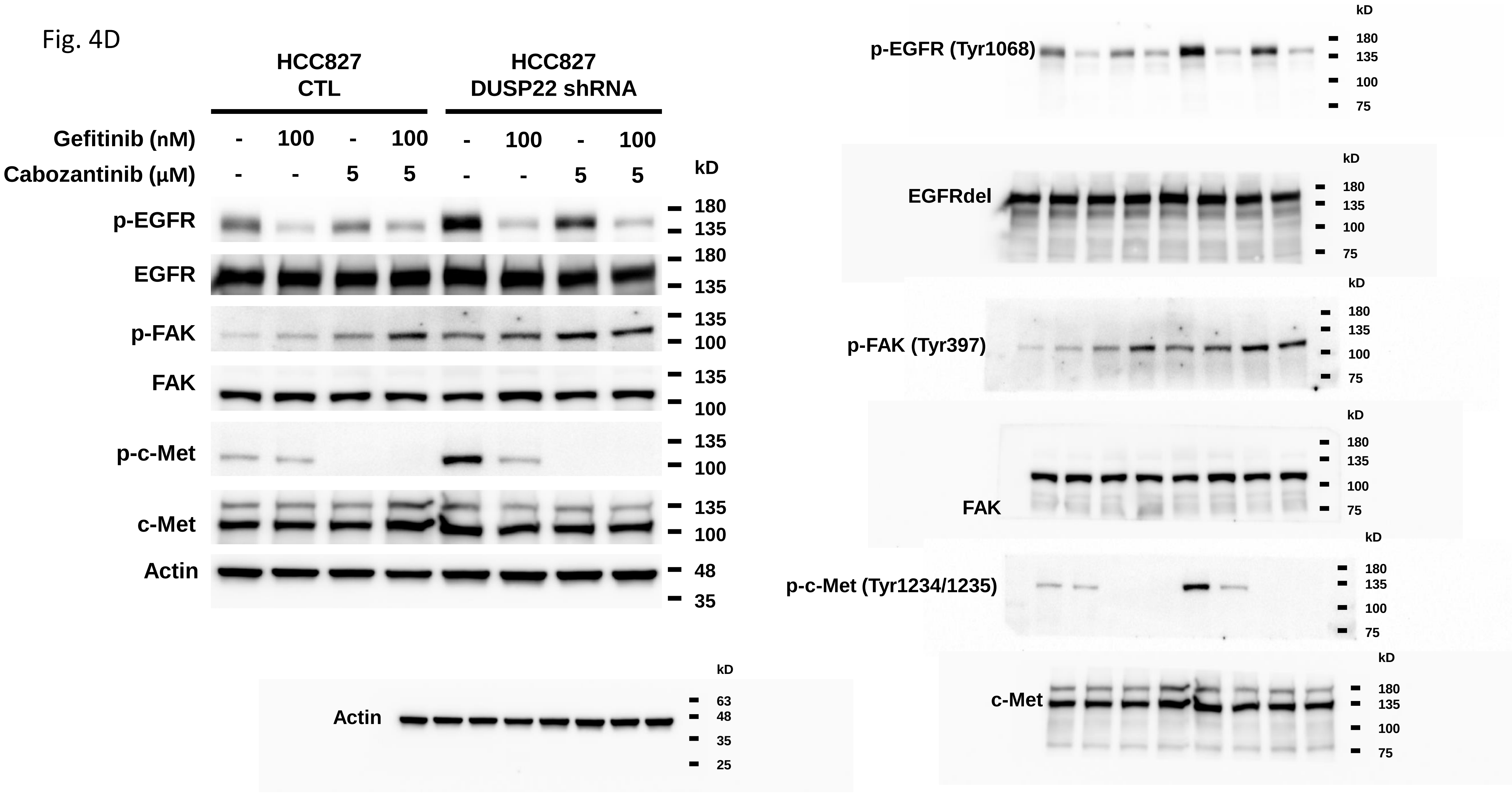
HCC827
CTL
HCC827
DUSP22 shRNA
Gefitinib (nM) - 100 - 100 - 100 - 100
Cabozantinib (µM) - - 5 5 - - 5 5
kD
p-EGFR
EGFR
p-FAK
FAK
p-c-Met
c-Met
Actin
180
135
100
48
35
p-EGFR (Tyr1068)
EGFRdel
p-FAK (Tyr397)
p-c-Met (Tyr1234/1235)
63
75
25

Fig. 5A left panel

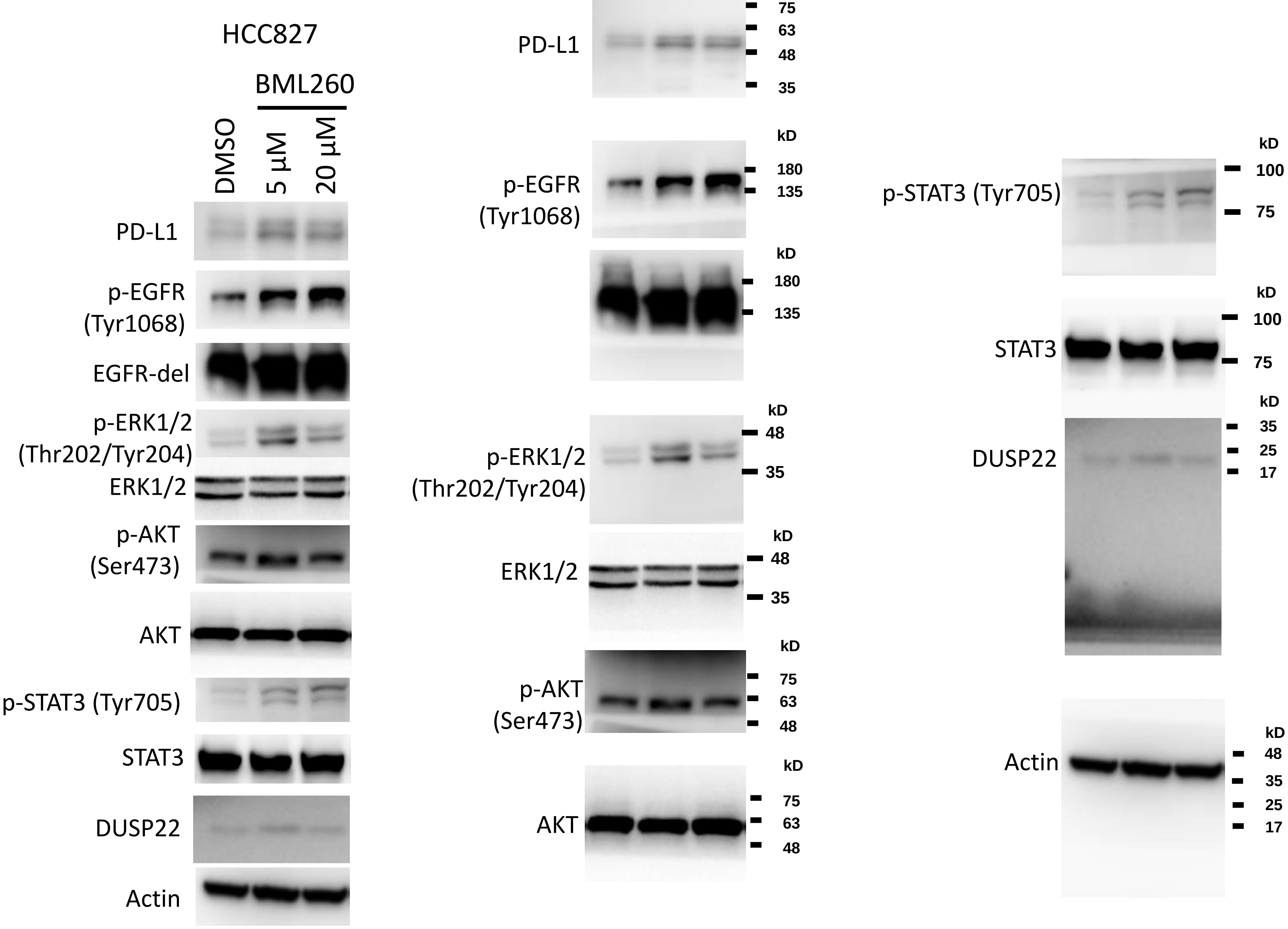

Fig. 5A right panel

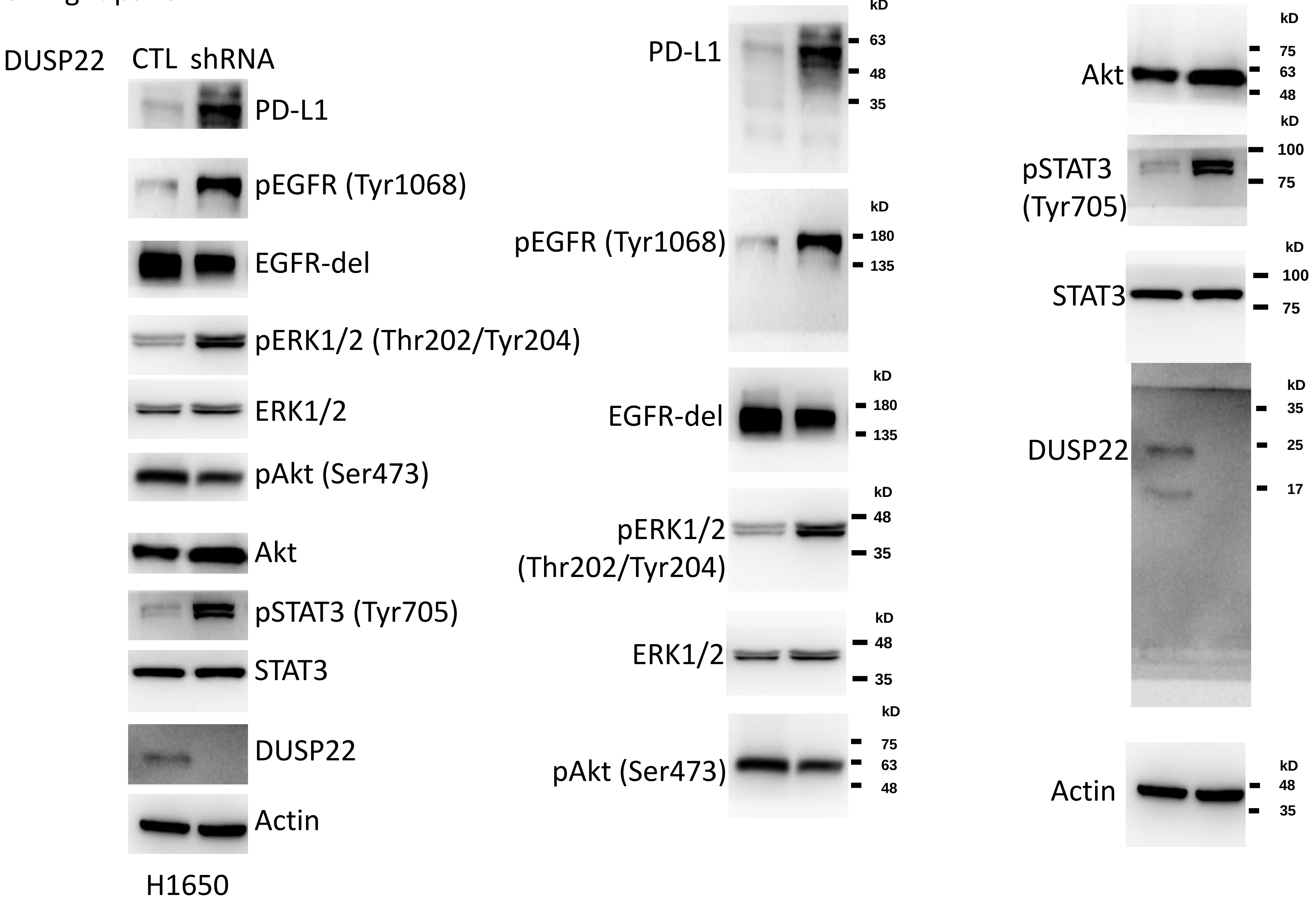

Fig. 6A

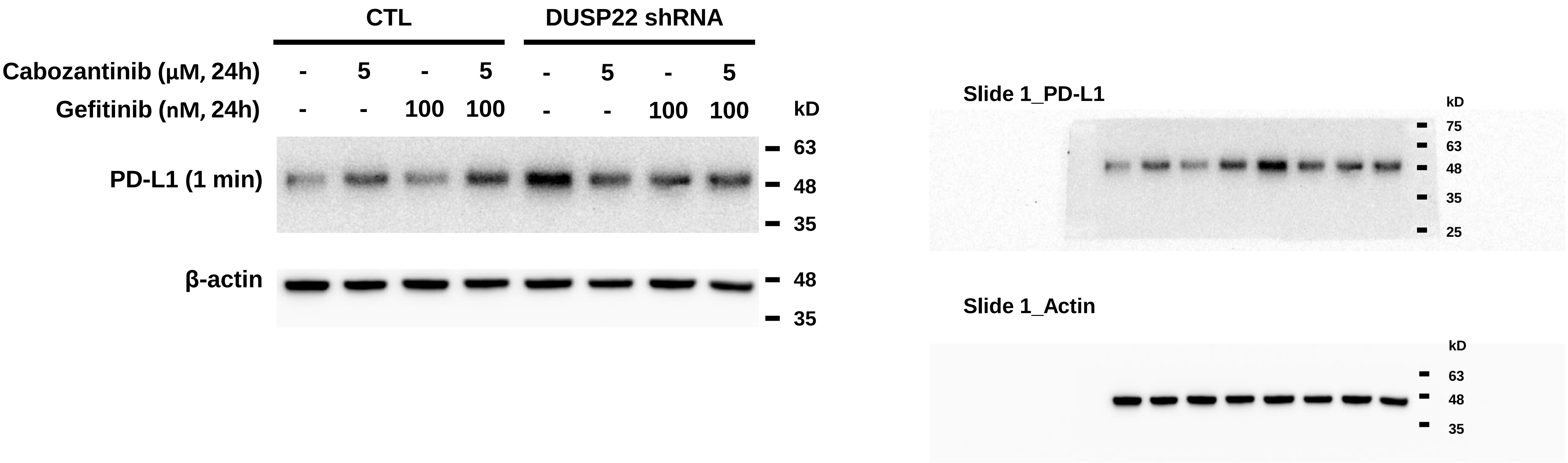

CTL
DUSP22 shRNA
Cabozantinib (μM, 24h) - 5 - 5 - 5 - 5
Gefitinib (nM, 24h) - - 100 100 - - 100 100
kD
PD-L1 (1 min)
63
48
35
β-actin
48
35
Slide 1_PD-L1
kD
75
63
48
35
25
Slide 1_Actin
kD
63
48
35

Supplementary Fig. 2A

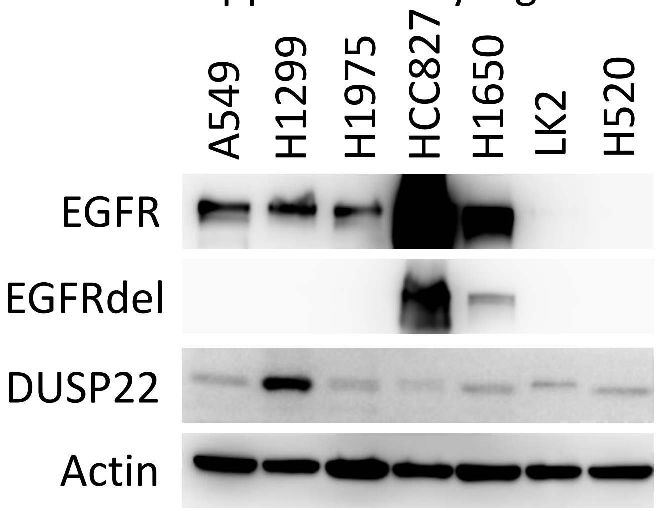

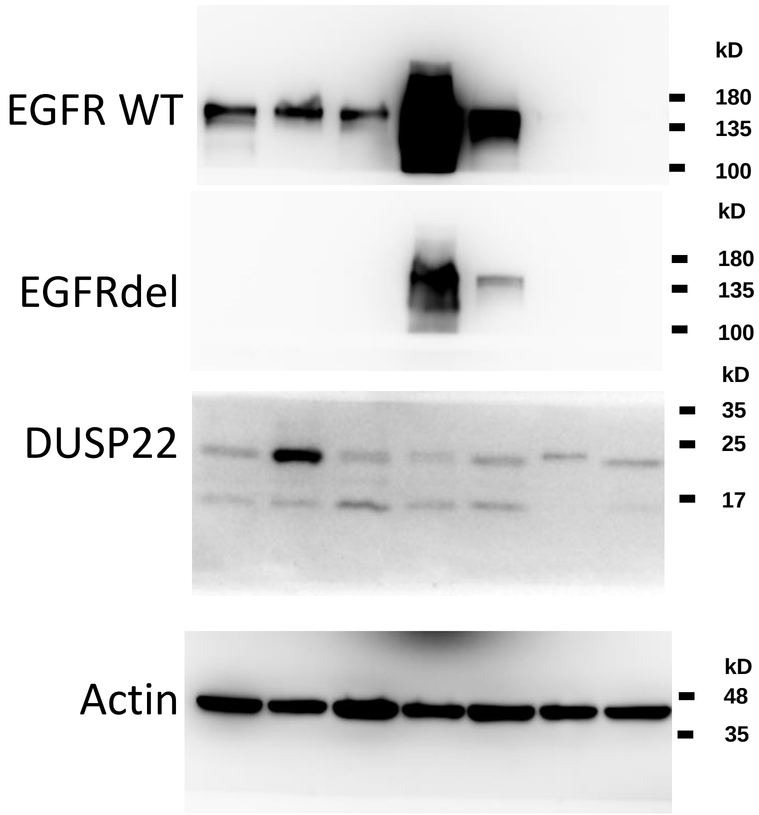

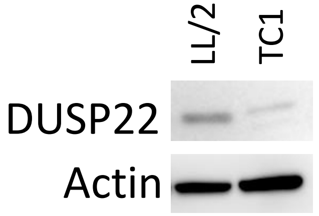

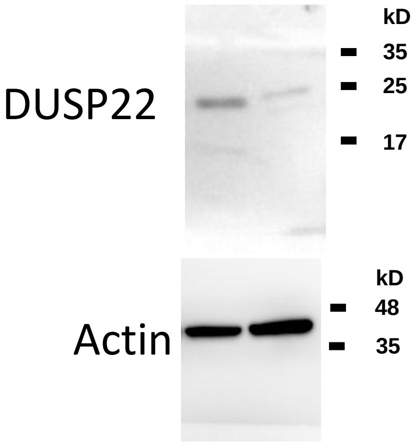

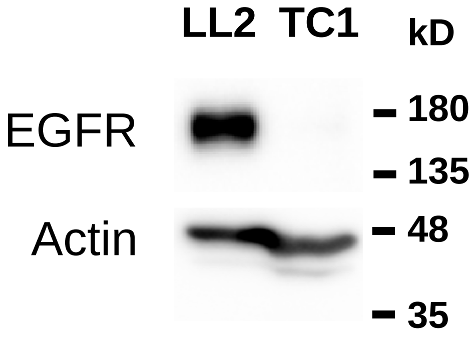

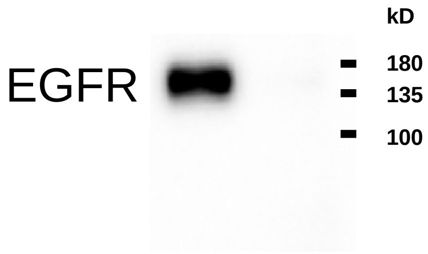

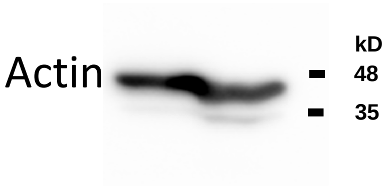

Supplementary Fig. 2B

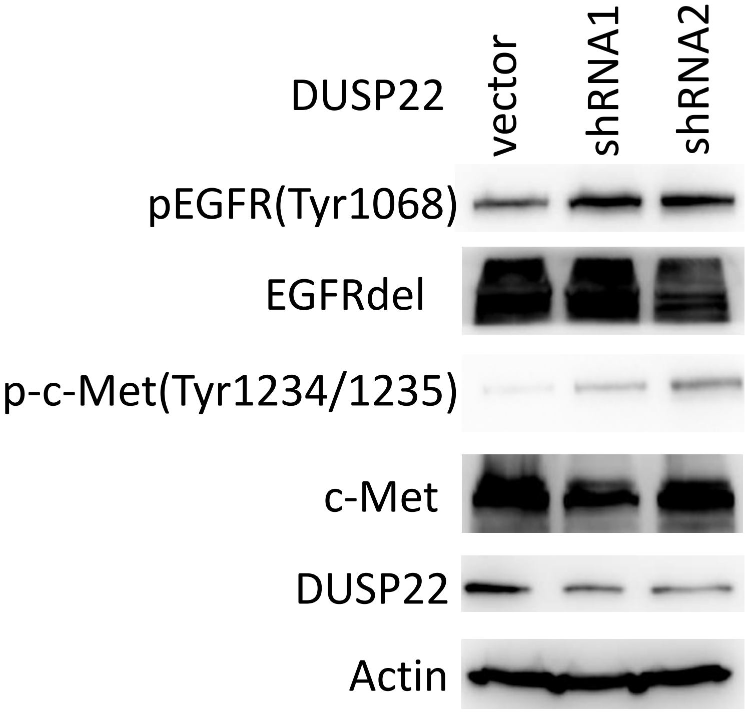

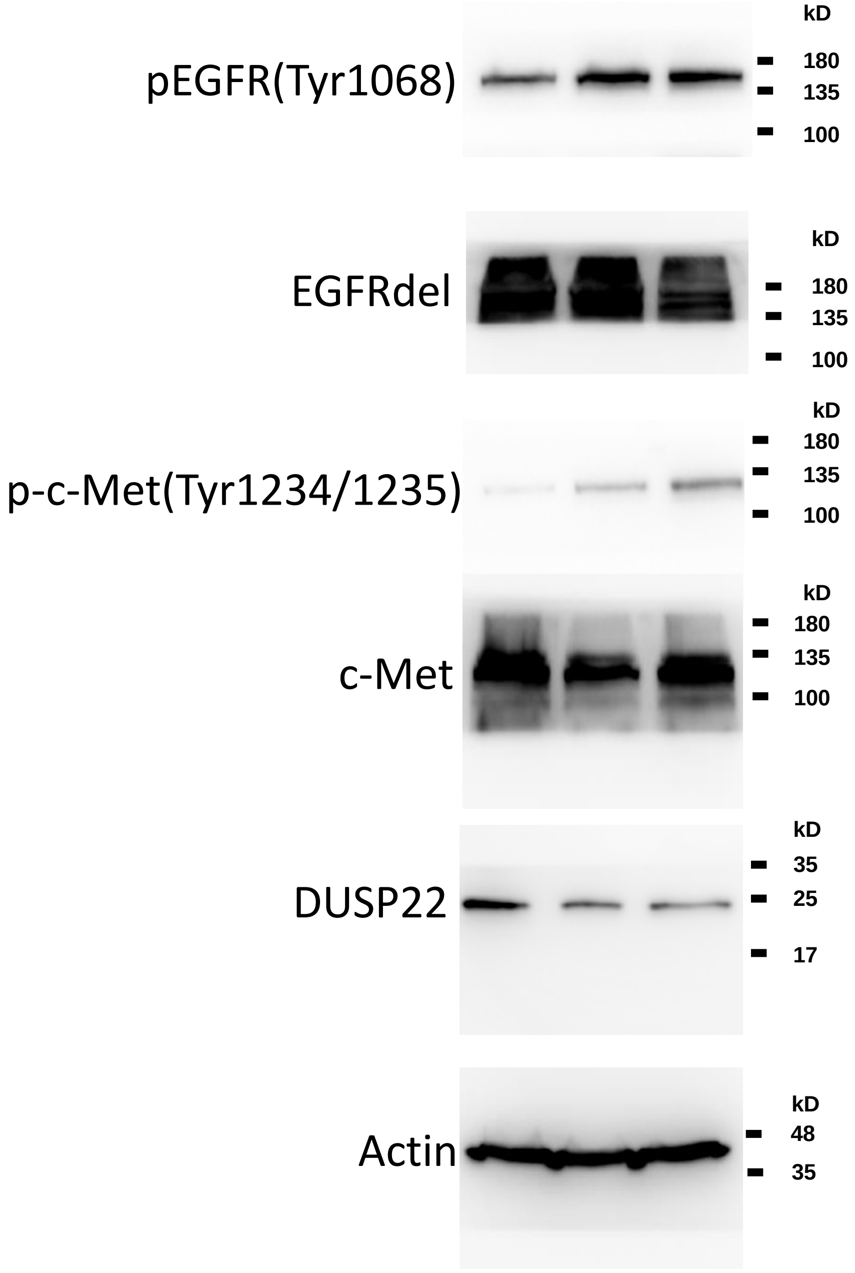

Supplementary Fig. 2C

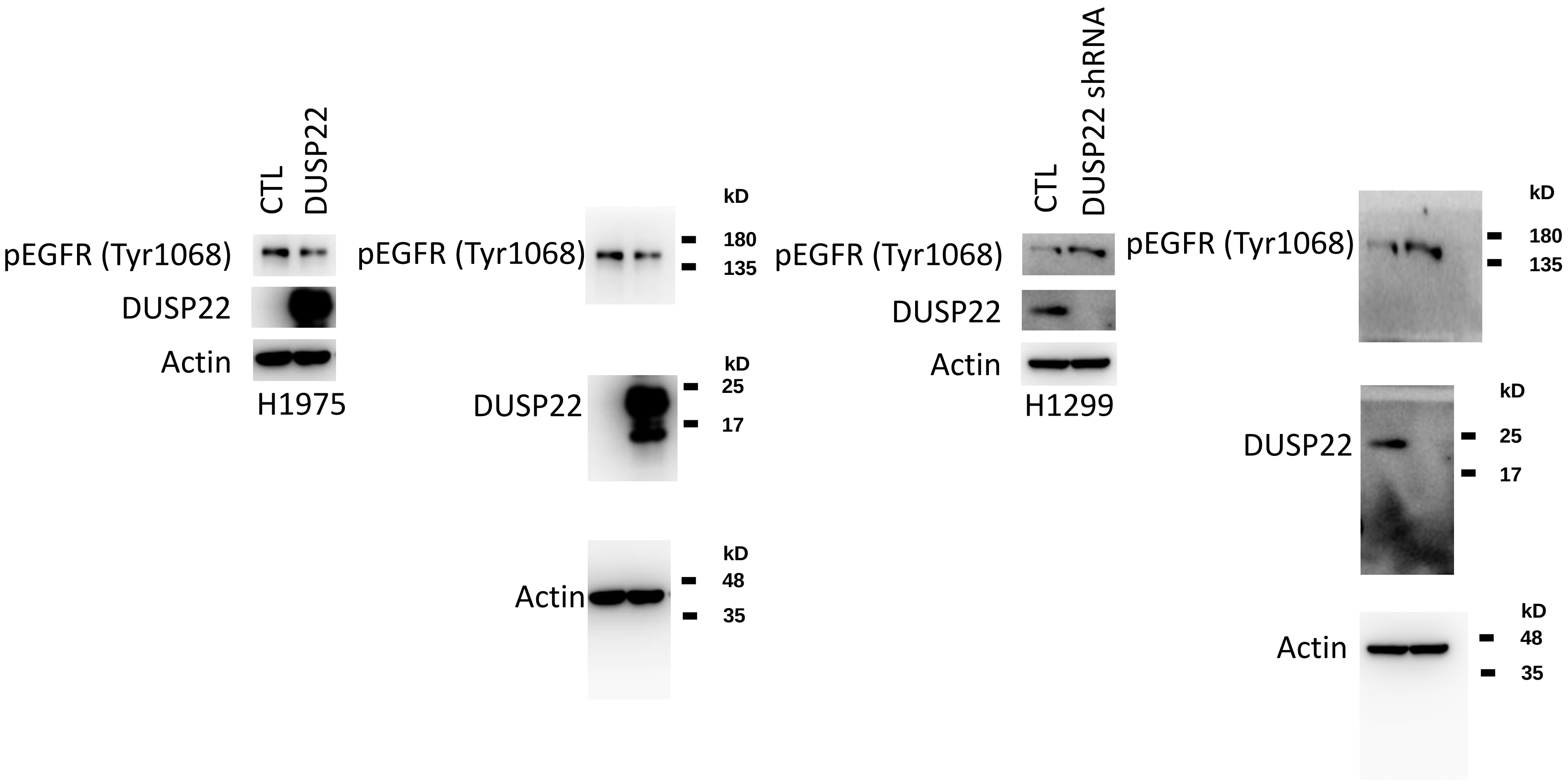

Supplementary Fig. 2E left panel

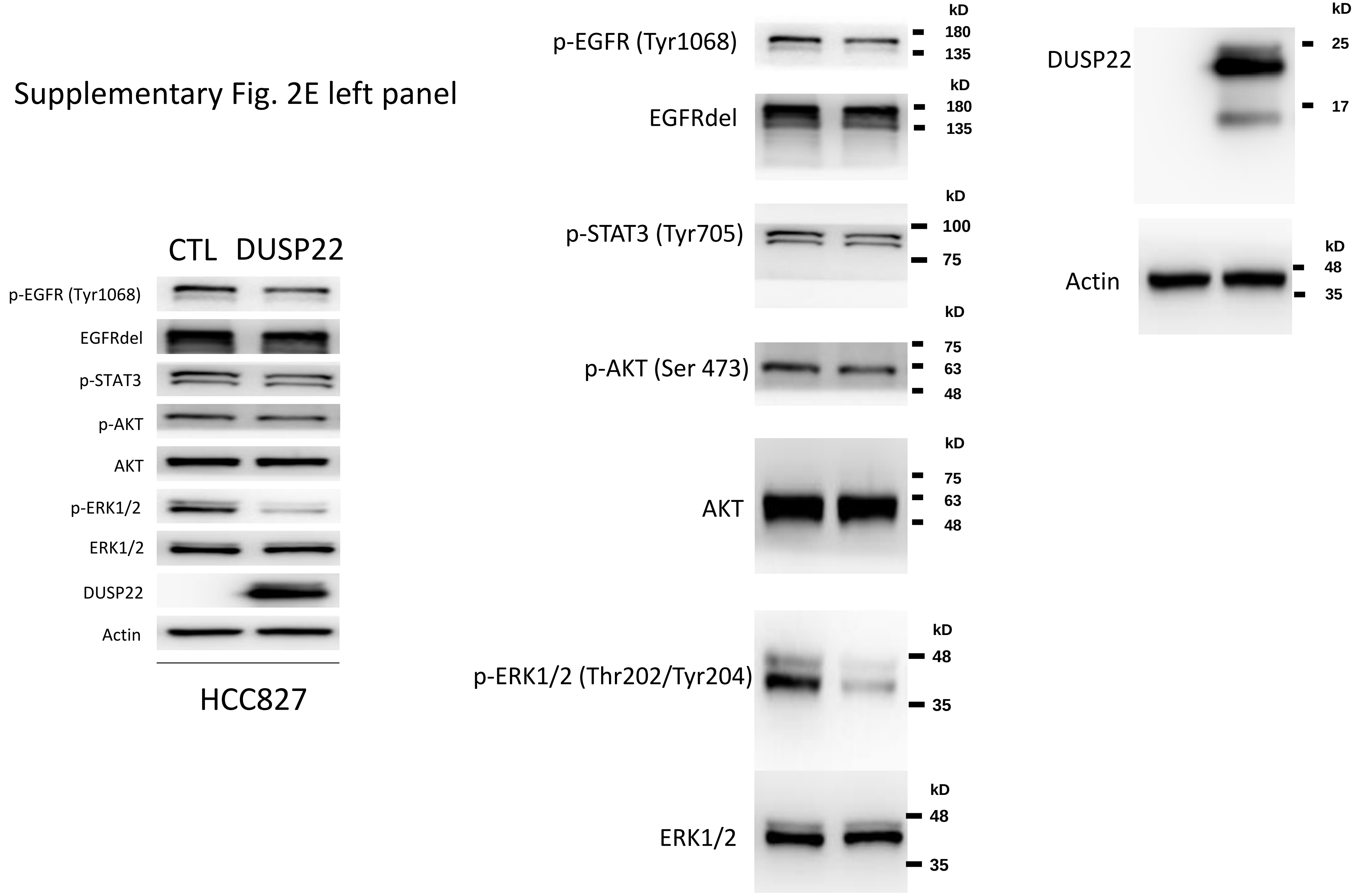

Supplementary Fig. 2E right panel

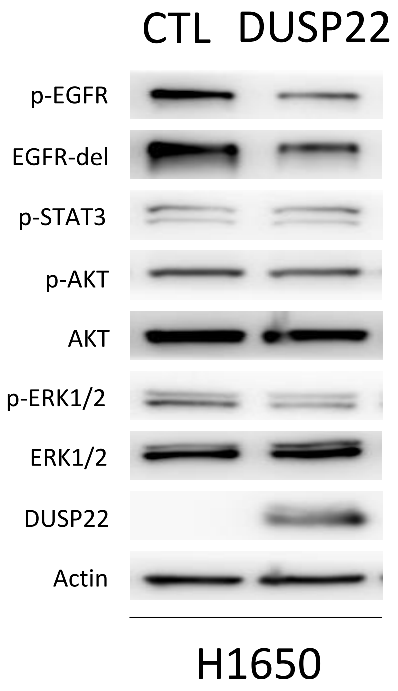

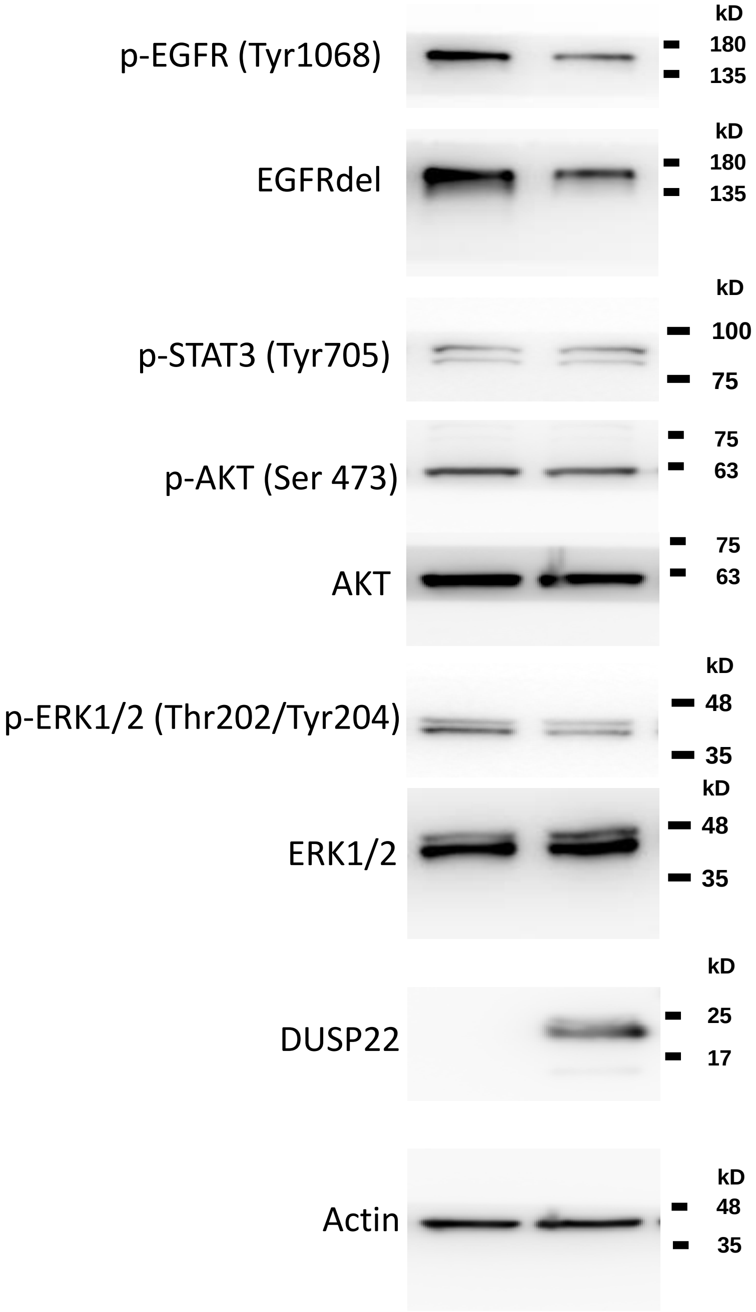

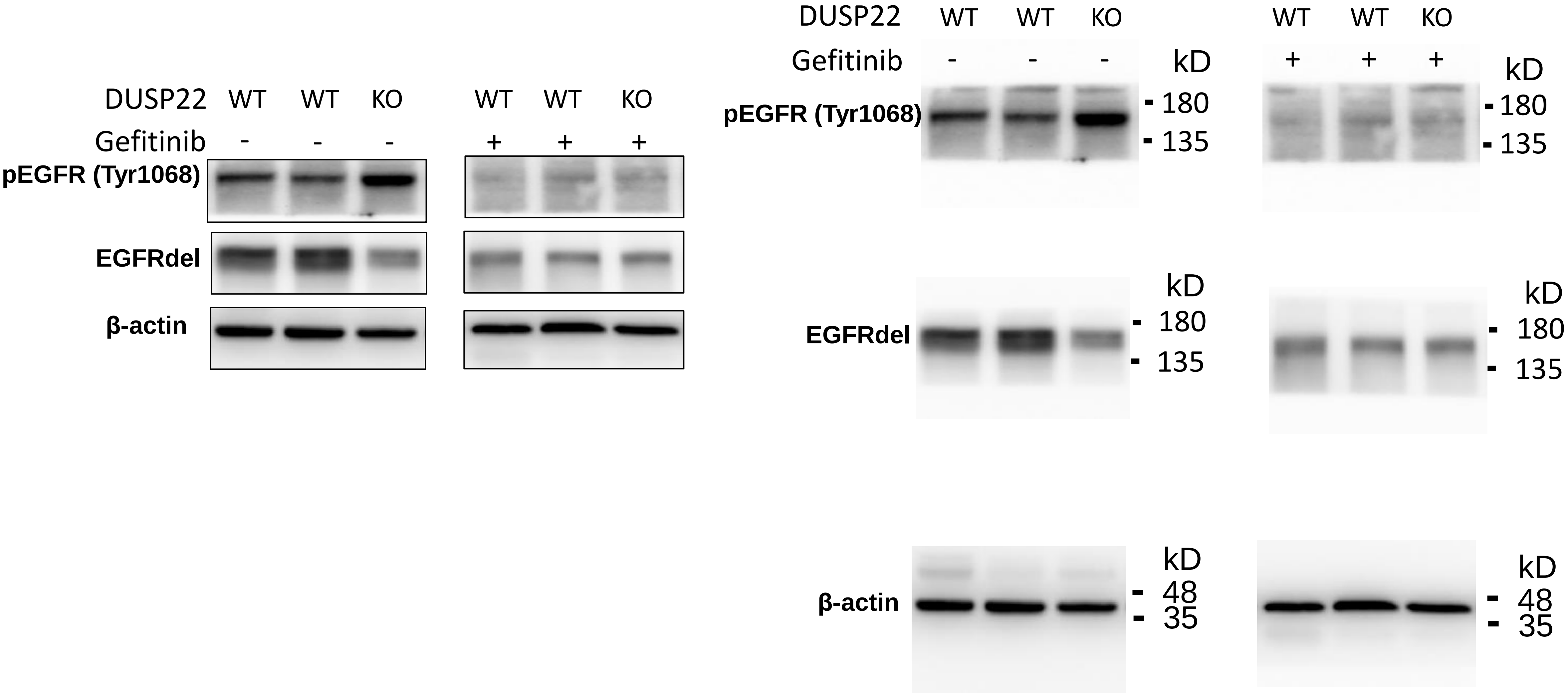
DUSP22 WT WT KO WT WT KO
Gefitinib - - - + + +
pEGFR (Tyr1068)
EGFRdel
β-actin
DUSP22 WT WT KO WT WT KO
Gefitinib - - - + + +
pEGFR (Tyr1068)
kD
180
135
EGFRdel
kD
180
135
β-actin
kD
48
35

Supplementary Fig. 4A

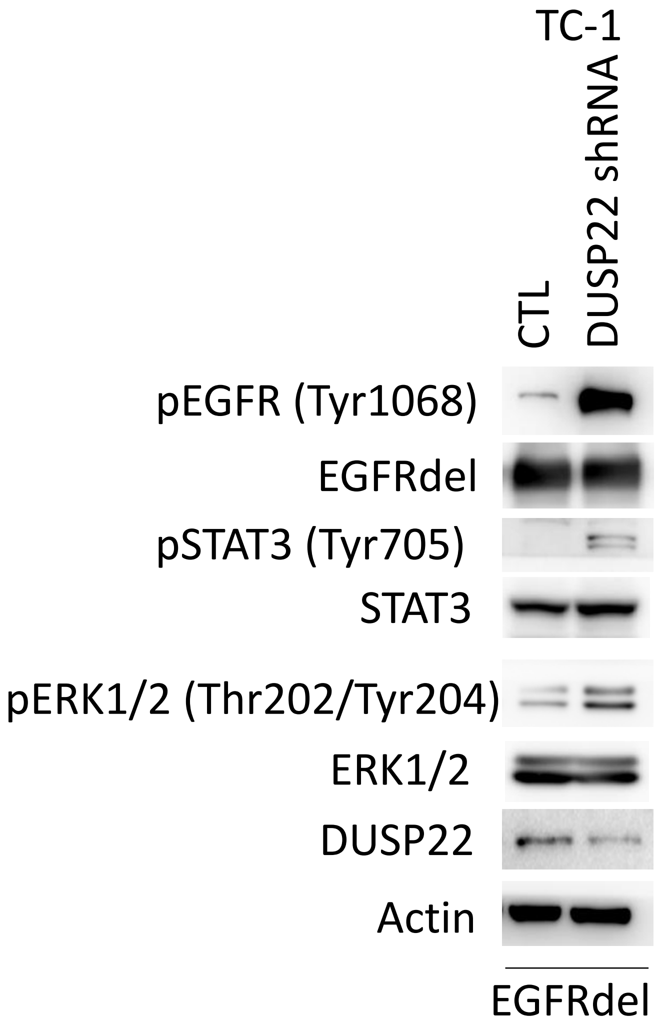

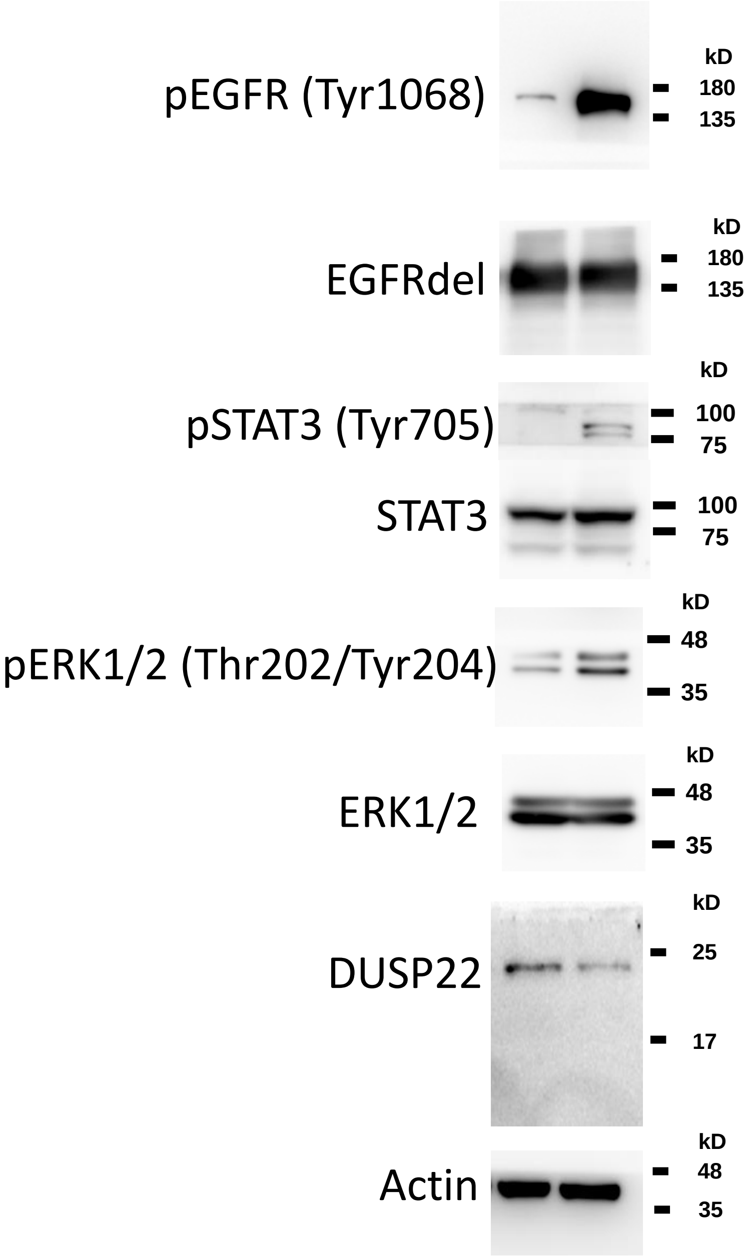

Supplement: Supplementary file 3 — Raw image file of all Western blot images [file 41420_2024_2038_MOESM3_ESM.pdf]
